# Developing a research agenda on NATure-based and Animal-assisted Intervention Strategies (NATAIS) in people with neurodegenerative diseases with a specific focus on social isolation and loneliness: a group concept mapping procedure

**DOI:** 10.1186/s12877-024-05387-2

**Published:** 2024-09-28

**Authors:** I. J. N. Declercq, R. Leontjevas, M.-J. Enders-Slegers, M. Molog, D. L. Gerritsen, K. Hediger

**Affiliations:** 1 Department of Primary and Community Care, Radboud University Medical Centre, Nijmegen, The Netherlands; 2 Faculty of Psychology, Open University of the Netherlands, Heerlen, The Netherlands; 3 Faculty of Psychology, University of Basel, Basel, Switzerland

**Keywords:** Neurodegenerative diseases, Dementia, Loneliness, Social isolation, Animal-assisted Intervention, Green care, Research gaps

## Abstract

**Background:**

Social isolation and feelings of loneliness are very prevalent in people with neurodegenerative diseases and are associated with a lower quality of life and other negative outcomes. These problems were increased during the COVID-19 pandemic resulting in initiatives to address social isolation. Given the potential benefits of nature-based and animal-assisted intervention strategies (NATAIS), it is crucial to further investigate if and how these strategies might minimize negative effects of social isolation and feelings of loneliness in this population. Therefore, the aim of this project was to develop a research agenda for NATAIS in people with neurodegenerative diseases, especially during challenging times, such as pandemics.

**Methods:**

This article outlines the process and results of a group concept mapping procedure aimed at developing a research agenda based on a logic model. In total, 19 work group members participated through a combination of in-person and online group meetings. Additionally, face-to-face group sessions were held at two international scientific conferences, during which feedback was solicited from 12 experts in the field of NATAIS and psychogeriatrics.

**Results:**

The group concept mapping procedure resulted in 14 clusters describing various future research topics, which were further refined and detailed during group discussions. The remaining eleven clusters, encompassing important research themes within the field of NATAIS, were organized into a logic model and summarized into the research agenda. The overarching cluster ‘ethical issues, possible risk factors, and their solutions’ was considered the most relevant during times of increased social isolation, such as during a pandemic, along with the necessity for more accessible NATAIS.

**Conclusions:**

This project resulted in a research agenda, directing future research and fostering collaboration between practitioners and researchers in the field of NATAIS. Such an enhanced partnership between science and practice has the potential to significantly contribute to the well-being of people with neurodegenerative diseases, in their daily lives and also during pandemics.

**Supplementary Information:**

The online version contains supplementary material available at 10.1186/s12877-024-05387-2.

## Background

Mood disturbances such as symptoms of depression, a lower quality of life, and an increased all-cause mortality are some of the many negative outcomes associated with social isolation and loneliness [1–3]. Social isolation is defined as the objective lack of social contact or support, while loneliness is the rather subjective feeling of being alone or isolated [4, 5]. Due to deteriorating health and socio-demographic changes such as the loss of a spouse, reduced social resources, and transition to a long-term care facility, people with neurodegenerative disease (PwND) are at higher risk to become socially isolated and to experience loneliness [5, 6]. Because of anti-pandemic measures like lockdowns, issues related to social isolation and loneliness became more acknowledged determinants of mental health and well-being [7, 8]. Therefore, there is a need for continued research on interventions targeting loneliness and social isolation.

Nature-based and animal-assisted intervention strategies (NATAIS), have the potential to reduce loneliness and social isolation [5, 9]. This paper refers to NATAIS as a variety of strategies that may encompass different activities in nature, indoor and outdoor interactions with animals or plants. Such strategies can be part of structured programs that align with green care principles, i.e., interventions, examples are horticulture therapy and green exercise [10]. In addition, NATAIS include non-therapeutic and informal strategies that can enhance individuals’ well-being, such as gardening, and interacting with pets. These strategies may provide opportunities for individuals to experience a sense of meaning and pleasure.

Research on nature-based interventions suggest that these interventions may address social connectedness [9], and promote active and meaningful community-life [11], which are seen as key features of successful interventions to reduce social isolation and loneliness [5]. Strategies used in these interventions like park visits, gardening, or bird watching are among the many nonpharmacological approaches often used to enhance social activities [9, 12]. Moreover, qualitative studies indicate that PwND appreciate the natural environment, reporting feelings of pleasure, relaxation, enjoyment of nature while being in fresh air, and the appreciation for the sounds and smells they experience [13].

Regarding animal-assisted interventions, reviews and meta-analyses show that integrating animals into healthcare and therapeutic interventions for PwND can lead to improvements in psychosocial, cognitive, and behavioral domains [14, 15]. These interventions may also reduce social isolation and loneliness [5], and enhance the quality of life for PwND [16]. In addition, since pets provide companionship, social support, and reduce feelings of loneliness [17, 18], pet keeping potentially diminishes the negative effects of social isolation by reducing feelings of depression, anxiety, isolation, and loneliness [19]. Qualitative research on this topic supports these hypotheses on reduced feelings of loneliness and other negative outcomes related to social isolation [20]. The positive effects of being a pet keeper persisted during the COVID-19 pandemic, and were attributed to the social interaction during outdoor walks, perceived social support, and the provided structure from pet-related responsibilities [20–22]. Yet, at the same time, challenges, such as difficulties in meeting pets’ needs, were exacerbated during the pandemic [23].

Although research shows positive effects of NATAIS in reducing social isolation and loneliness, reviews often conclude that the quality and currently-available evidence is limited and more research is needed to assess the (cost) effectiveness and positive effects, particularly in PwND and their caregivers [5, 24, 25]. Furthermore, several studies on the effects of NATAIS on loneliness and social isolation have found no positive effects [26–28]. Another challenge is the fragmented character of NATAIS research. While it seems reasonable to integrate both nature and animals in research (animal-assisted interventions can take place in a natural environment, and nature-based activities can involve animals), research in this area is currently divided into two largely independent fields. Research groups tend to focus either on nature-based activities or on animal-assisted interventions. This division has led to uncertainty about which elements of NATAIS are responsible for possible effects.

The objective of this project was to develop a research agenda aimed at stimulating and guiding research on the effects of NATAIS for PwND and their caregivers during times of increased social isolation, such as pandemics like COVID-19. Next to (1) setting the vision, mission and potential research questions and interventions, this project aimed to (2) identify the objectives and conditions necessary to achieve success in future research on this topic.

## Methods

**Fig. 1:**
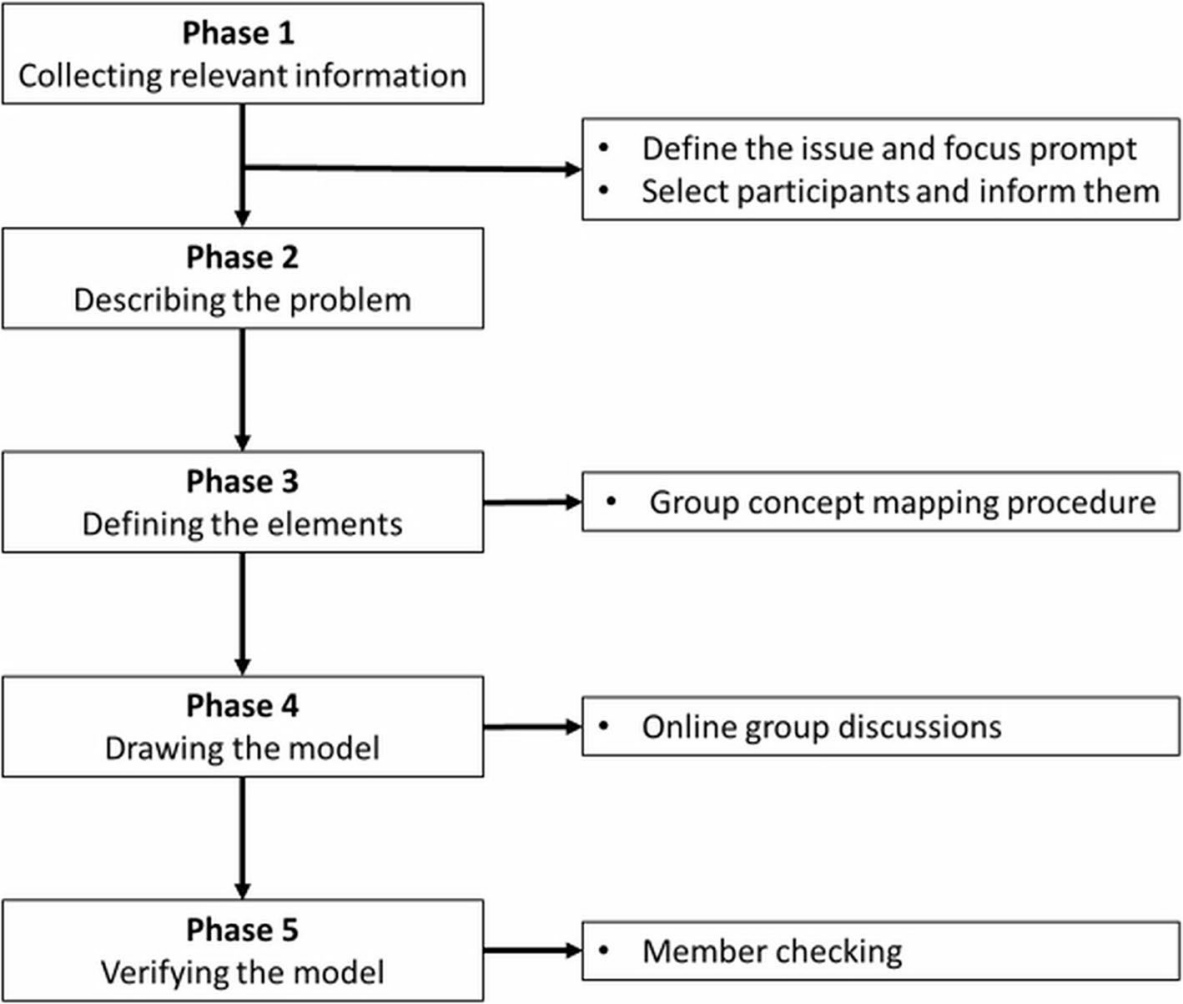
Workflow of the project (based on McLaughlin & Jordan, 2015)

The aim of this project was to develop a NATAIS research agenda addressing social isolation in PwND. A logic model was used [29, 30] to frame potential research questions into a research agenda. A logic model is a valuable tool for developing research programs, as it can help clarify research questions, theoretical hypotheses, and identify the most important research elements, such as variables and outcomes to measure [31]. It organizes these important elements, and assists in designing and conducting research and implementation evaluations, which are relevant in daily practice and for various stakeholders [30]. Input for the logic model was given through an online group concept mapping method (GCM), using the Concept Systems Global MAX^tm^ software [32]. GCM is a participative method that facilitates a group of stakeholders from different communities of interest, to reach a shared vision regarding a particular issue online, independently and at their own pace in an objective way [33–35]. This method was chosen as it enables to seek information in different populations, integrating perspectives and variation in stakeholder knowledge and opinions regarding the topic [34]. The procedure used in this project is visually presented in Fig. 1.

### Defining the problem (phase 1 and 2)

In the first two phases, the core team members outlined, through discussion, the problem that needed to be addressed in the research agenda. This discussion was then summarized into a focus prompt, enabling to maintain focus on the topic during the brainstorm phase. The core team guided the entire process and included experts in gerontology (RL and DG), anthrozoology (KH and MES), and two PhD students (in gerontology, ID and in anthrozoology, MM). To ensure the perspective of different stakeholders, an international and interdisciplinary working group was constructed, including both academic staff (i.e., researchers and lecturers, both in NATAIS and care for older adults) and practitioners of NATAIS (i.e., policy associates, veterinarian advisor and dog trainers, psychologist, coaches and nurse specialists). Next to the core team and a representative of the university that coordinated the project financially, 14 additional working group members were recruited within the network of the core team members. These working group members were briefed about the project through an information letter and during an online meeting, where aims and methods of the project were also further discussed.

### Defining the elements within the logic model using the GCM procedure (phase 3)

#### Online brainstorming

During phase 3, working group members were first asked to generate research ideas or questions for research (further referred to as research ideas). The ideas were collected using an online platform, namely GroupWisdom [32]. Each group member independently added as many potential research ideas as possible using the following focus prompt: “*Based on your experience, what areas of research are relevant in everyday life and in therapeutic context for people with neurodegenerative disorders during times of social isolation (such as during COVID-19 pandemic restriction)?”.*

#### Data collection

Second, to maintain a consistent set of unique ideas, the collected research ideas were split up, aggregated, removed, and/or slightly edited according to the synthesis process outlined by Kane & Trochim [34]. A maximum of 100 statements was set as the threshold [34]. This process of idea synthesis was conducted by two researchers of the core team and resulted in a reduced set of unique statements that contained all the research ideas collected by the various working group members.

Third, all working group members were invited a second time to complete the next steps (i.e., sorting and rating) online in GroupWisdom. Each working group member was asked to individually sort the reduced set of statements in a way that made sense from their own perspective. In addition, working group members were asked to rate these statements on relevance, based on their own perception and experience regarding importance in times of social isolation, for example, during a pandemic. To rate the statements, a 4-point Likert scale was used (1 = *not relevant during pandemics*, 4 = *very relevant during pandemics*).

#### Analysis and interpretation

Fourth, the collected data were further analysed and interpreted. To ensure data quality, the collected data were reviewed for completeness and random answering strategies. For a respondent’s input to be included in the analysis, data had to be sorted or rated for at least 75% of the statements [32]. Further analysis included the construction of a similarity matrix and multidimensional scaling procedures. This was facilitated with the Concept Systems Global MAX^tm^ software [32, 34]. Proximity data, i.e., similarities/dissimilarities between statements across all participants [34, 36], were further quantified using a nonmetric multidimensional scaling procedure. This process assesses iteratively the distance between items in a two-dimensional solution, and represents the “perceived similarity between items relative to other items in the space” [36]. The process resulted in a set of X–Y values, plotted in a point map [34, 36]. The level of agreement between the estimated X–Y values and the inputted data proximity was expressed in ‘stress-values’, which usually range from 0.205 to 0.365 [34]. The lower the stress-value, the better the model fits the data [36].

#### Development of the research agenda

Finally, using the calculated X–Y values, hierarchical cluster analysis was performed to group items into clusters [34]. Different solutions were compared to each other, considering bridging values and the content of the clusters and their included statements. The resulting most suitable solution was further discussed and finalized during an in-person meeting with the working group members. During this meeting, the name, content, and included statements of the clusters were discussed in subgroups. Disagreements with the proposed clusters were further discussed plenarily and, if required, adapted. Each cluster, resulting from these group discussions, was considered as one theme of the research agenda. Subgroups, consisting of three to four members, were formed to further summarize the current evidence and research gaps of the different clusters (i.e., themes) into the research agenda. The final version of the research agenda was edited by the core team members who requested and accommodated feedback from the working group.

Next to determining the above-mentioned themes based on the group concept mapping procedure, the in-person meeting of the work group was used to agree upon a uniform vision and mission and to discuss further dissemination and implementation plans.

### Drawing the model (phase 4)

The themes described in the research agenda were, during phase 4, used as key elements to further define the logic model. This model was further discussed by the core team members, and used to structure and organize the different themes within the research agenda.

### Verifying the model (phase 5)

The primary goal of the fifth phase was to obtain membership consensus on the constructed logic model and the finalized research agenda. All team members were asked to read the final version and provide feedback on the themes that are relevant to their professional interests. In addition, feedback from experts in the field of NATAIS and psychogeriatrics was solicited during two in-person meetings at conferences, once at the International Society for Anthrozoology congress 2023 in Edinburgh and once at the International Psychogeriatric Association congress 2023 in Lisbon. During these meetings, experts were encouraged to reflect on the developed agenda from the perspective of their own expertise and to further discuss the necessary conditions to achieve success for future research on this topic.

## Results

### Work group team members

Out of the 21 invited NATAIS work group members, 19 completed the online participant questions, 15 (78.95%) had a background in research, often combined with a practicing role in various healthcare settings. Moreover, most of the members (N = 11) had over ten years of experience in topic specific areas, three members had between 5 and 10 years of experience, four members had between 1 and 5 years, and one member had less than a year of experience. An overview of the contributors, and their expertise, can be found in the colophon on page 2 of the research agenda (See Additional file 1).

### Outcome of the GCM procedure (phase 3)

In total, 12 out of 21 invited work group members generated 104 raw research ideas, resulting in 127 unique research ideas. After the aggregation procedure and removing duplicates, 90 ideas remained and were reformulated into unique statements. Although we started with a specific pandemic-related prompt, the responses extended beyond just pandemic situations. Therefore, the research agenda was expanded to encompass research themes in broader contexts.

Further clustering and rating of the statements were conducted by 17 out of 21 invited work group members, from which one member was excluded because only 24% of the procedure was completed. This clustering procedure resulted in a point map with stress-value of 0.27.

**Fig. 2:**
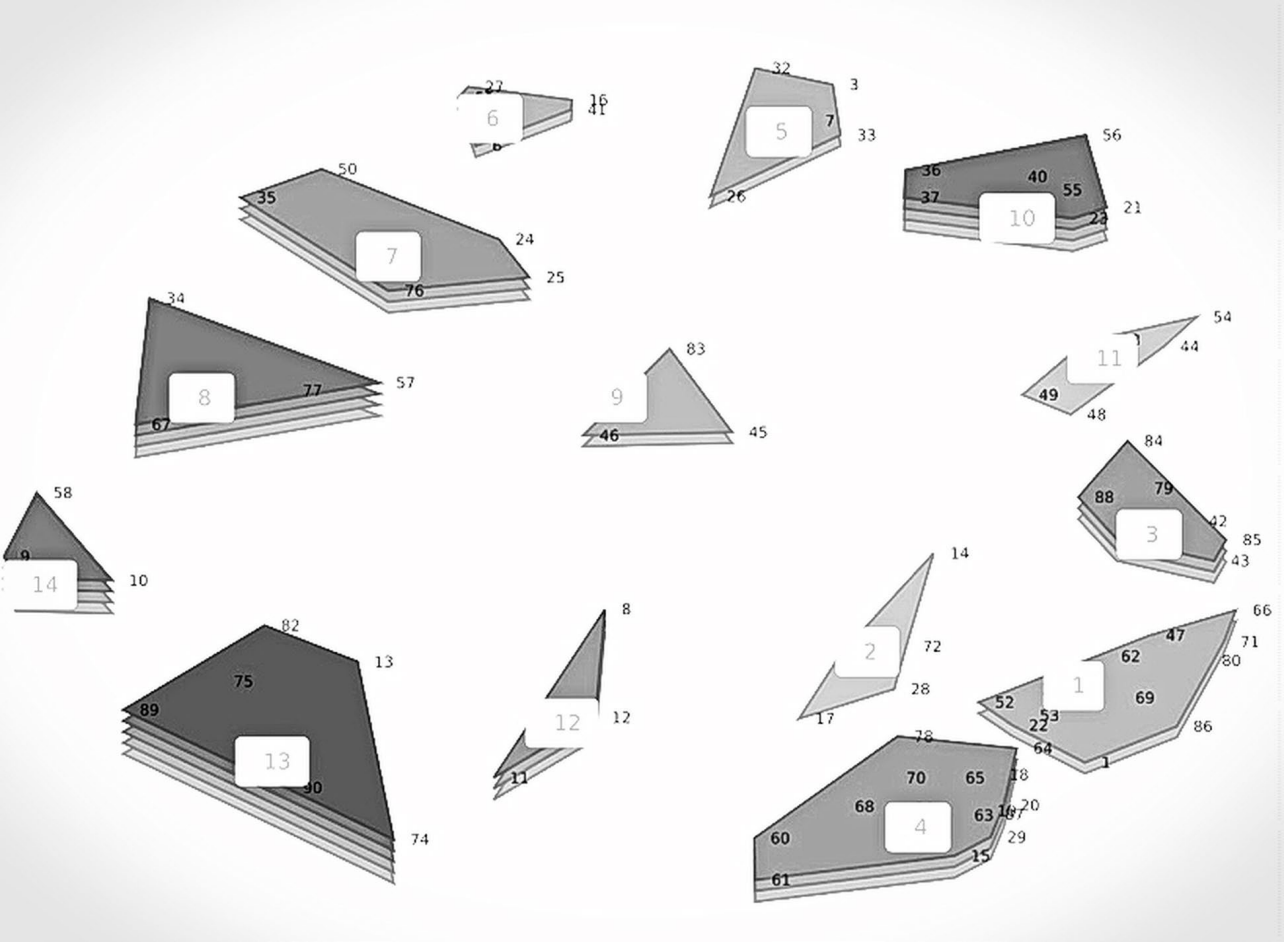
Result of the multidimensional scaling produce: The cluster solution rating map. The numbers displayed on the map, represent the 14 original clusters, along with all unique statements (see Additional File 2, Table 1). The number of layers represent participants’ rating values of the relevance of the clusters during pandemics with values ranging from 2.50–2.62 for layer 1 and 3.00–3.13 for layer 5

**Fig. 3:**
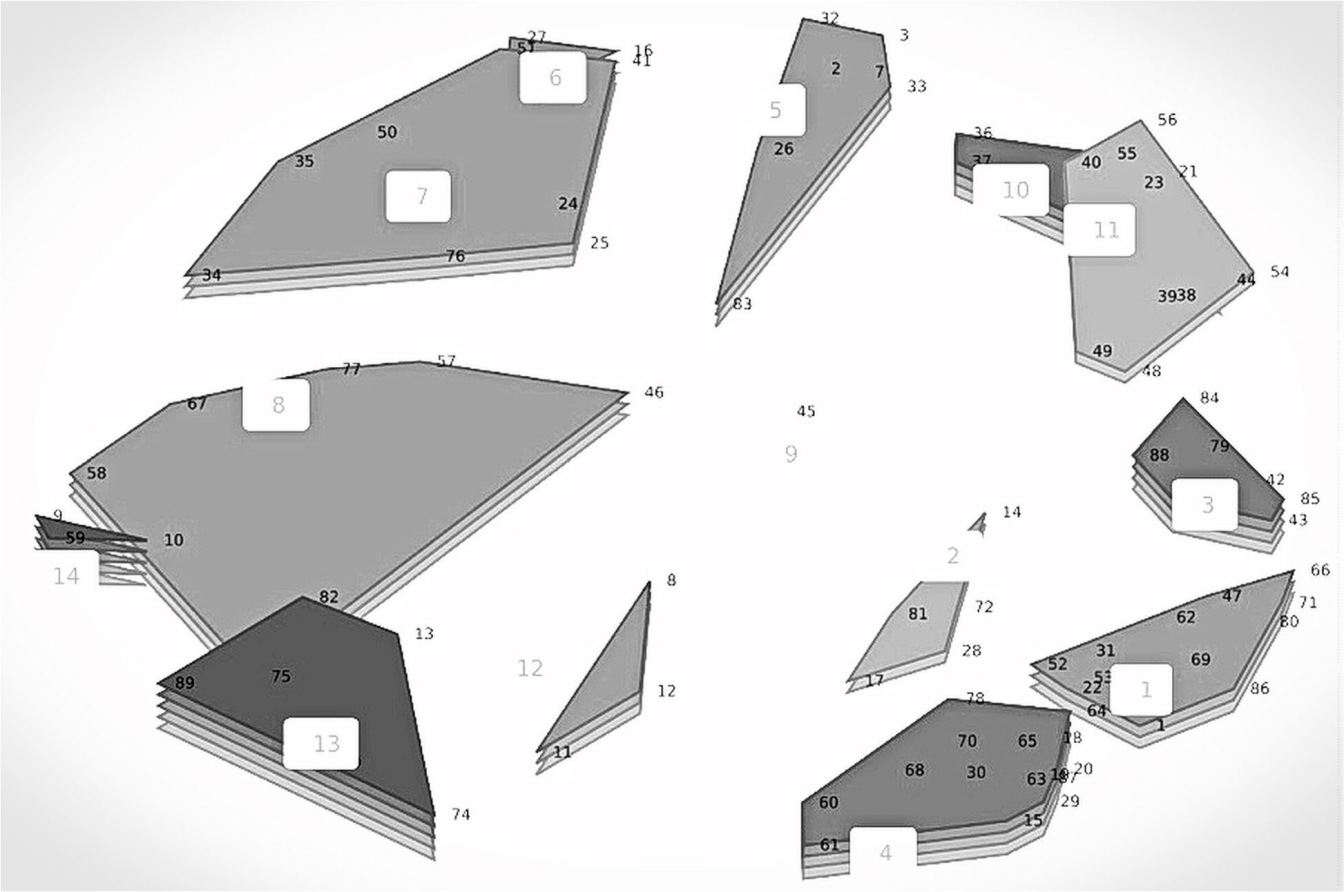
Result of the group discussion: Novel cluster solution rating map. The numbers displayed on the map, represent the 14 novel clusters, along with the reorganized statements (see Additional File 2, Table 1). The number of layers represent participants’ rating values of the relevance of the clusters during pandemics with values ranging from 2.27–2.45 for layer 1 and 3.02–3.21 for layer 5

The 14 cluster solution map was considered the best option for the initial version of the agenda and was further discussed during the in-person meeting (see Fig. 2). Although 14 clusters remained during this discussion, some adjustments were proposed and consented during the plenary group discussion (see Fig. 3). A detailed overview of the initial statements, together with the cluster names and outcome of this group discussion, can be found in Additional File 2.

The cluster describing ‘Ethical issues’ was found to be the most relevant during pandemics. The second most relevant themes during pandemics were ‘Research into accessible NATAIS’ and ‘Technological solutions’ (see Figs. 2 and  3).

### Development of the research agenda and logic model (phase 3, 4 and 5)

**Fig. 4:**
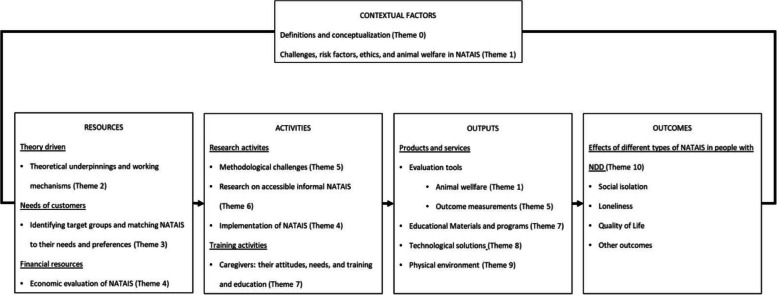
Logic model based on the identified themes

Subgroups of the meeting’s participants refined the clusters, resulting in ten themes that were summarized into a research agenda and further discussed with experts in the field. Since the participants and experts at the conference meetings stressed the importance of consensus on important concepts within NATAIS, ‘Theme 0—Conceptual research on NATAIS’ was added. In addition, participants drew attention to the difficulty of generalizing results within research (e.g., due to the differences within the variety of NATAIS, cultural differences, etc.), and provided additional advice on structuring and organizing the research agenda (see Fig. 4).

### Vision and mission

The vision and mission of the research agenda were discussed during the in-person meetings. Regarding the vision, the team members agreed that, to enhance the quality of life for PwND, it is important to recognize their right to have access to NATAIS. However, the available NATAIS activities are limited and mostly inaccessible for PwND. To address this concern, there is a need for expanded research focusing on improved knowledge and understanding of the applicability and accessibility of NATAIS. To achieve this, it was agreed that: 1) the primary focus of the agenda should be on long-term care, including both institutionalized and home care, 2) ethical issues, risks, and solutions should always be considered and balanced when conducting research on and practicing NATAIS, 3) extra attention should be given to diversity and cultural differences in PwND and their ecosystem, 4) the collaboration between practice and research should be encouraged to achieve success in research.

## Discussion

This project developed a research agenda regarding NATAIS, for PwND and their caregivers in times of increased social isolation, such as during pandemics like COVID-19. Through an iterative process, 11 main themes were identified. Here, we discuss the research themes in the order of they appear in the research agenda, which was structured into a logic model.

### Vision, mission, and objectives to achieve success (contextual factors)

The research agenda aims to play a pioneering role in advancing both knowledge and practical applications of NATAIS for creating a world where PwND can enjoy an improved quality of life, and reduced social isolation through innovative, evidence-based interventions. This agenda seeks to raise awareness, to stimulate nursing facilities and to encourage policymakers, legislators, and funding agencies to invest in additional research on the potential benefits of NATAIS, which may contribute to the availability and accessibility of NATAIS. The primary focus of the agenda is on long term care, employing a One Health approach, to ensure ethical and balanced research, with a priority on the well-being of all involved [37]. Consequently, an overarching NATAIS theme regarding ‘ethical issues, potential challenges, risk factors and their solutions’ was considered the most relevant. This theme has been underscored by participants in our expert meetings, highlighting them as critical elements and contextual factors that should be consistently considered in NATAIS research and practice. Regarding the primary objectives to achieve success, the research agenda emphasizes the importance of interdisciplinary collaboration among researchers, practitioners, caregivers, and PwND to ensure a holistic approach to NATAIS. This collaboration serves as a bridge that may deepen our understanding of stakeholder needs, help to identify key barriers to readiness for change, enrich our evidence-based knowledge base, and facilitate the effective implementation of NATAIS [38, 39]. During the conference meeting, experts stressed the significance of achieving conceptual agreement on NATAIS and defining clear outcomes of interest. These aspects were considered, next to the improved awareness of policy makers regarding the subject and sufficient funding of research activities, important pre-conditions to successfully conduct and evaluate research in the field.

### Inputs

Inputs in the logic model refer to all the resources that might be needed to support the research. Theoretical underpinning of NATAIS, inventorying the needs of the different target groups of NATAIS, and an economic evaluation can be regarded as essential inputs [30].

#### Theoretical underpinning of NATAIS

Although several promising hypotheses and theories regarding NATAIS have been proposed, there is currently no comprehensive theoretical framework that could explain the potential effects of NATAIS for PwND, particularly concerning social isolation and loneliness. Research in this domain may address two facets of loneliness. A first facet focuses on the characteristics of loneliness, e.g., social, emotional, and existential loneliness [40], while the second facet delves into the chronicity of loneliness, encompassing incidental, transient, and persistent loneliness [41]. Depending on the type of loneliness, different underlying theories and mechanisms may come to the fore. For instance, it is reasonable that the socioemotional selectivity theory [42, 43], could be more pertinent to emotional loneliness. This theory implies that meaningful relationships are more important than the size of the social network. In the context of NATAIS, the attachment theory, often related to meaningful contact with animals and pets [44], could serve as a theoretical framework for mitigating emotional loneliness. Another example of a relevant theory regarding loneliness in PwND, is the social-evaluative threat theory. This theory addresses loneliness as a hypervigilance to social threats [45, 46]. Hyperarousal was found to be significantly correlated with emotional loneliness in PwND during the COVID-19 pandemic, while this association was not observed in their caregivers [47]. Since hypervigilance is more strongly associated with higher cortisol levels [45], and cortisol levels have previously been linked to persistent loneliness and cognitive decline [41, 48], hypervigilance could be one of the many important underlying mechanisms predicting loneliness in PwND. Within the context of NATAIS, the attention restoration theory could offer a theoretical framework for the beneficial effects of NATAIS in reducing this hypervigilance. The attention restoration theory [49] posits that natural environments may help to restore experiences by transforming the directed attention into an effortless attention. Although this theory was primarily suggested with respect to fatigue, it could also provide a theoretical framework for redirecting hypervigilance to social threats in PwND, for example in times of pandemics like COVID-19 with increased social isolation.

#### Needs of the different target groups

It is important to first identify the needs of the different target groups in order to evaluate how NATAIS can assist in this regard. With regard to the research agenda, three distinct target groups can be delineated.

First, the agenda focusses on PwND. Neurodegenerative disease is “an umbrella term for a range of conditions which primarily affect the neurons in the human brain and causes problems with movement (called ataxias), or mental functioning (called dementias)” [50]. While the literature supports the objective that social isolation and loneliness contribute to reduced cognitive abilities and, therefore, are important topics that need to be addressed in PwND [51–54], little information is available concerning prognoses and differences in outcomes among the various neurodegenerative diseases. Additionally, little research is available about the needs and preferences of PwND, and how certain interventions, including NATAIS, might be experienced differently in PwND. For example, it could be argued that certain NATAIS, like park visits as social prescription, may be more challenging to implement in practice due to ataxias in PwND. This may enhance feelings of dependency on others [55] and subsequently, result in reduced quality of life [56, 57].

Second, because of their significant contribution to the well-being of PwND, it is imperative to identify the needs of family caregivers. A mixed method systematic review revealed that family caregivers need accessible and tailored information to provide adequate care to their loved ones [58]. However, to the best of our knowledge, research on how to translate this into the context of NATAIS is non-existent. In addition, due to affected personality and behavior of PwND, caregiving tasks in neurodegenerative diseases might be more challenging than in other diseases. Family caregivers of PwND reported high levels of emotional and physical stress putting them at a high risk of developing depressive symptoms [59]. Therefore, family caregivers might also benefit from NATAIS.

Third, it is important to identify the needs of professional caregivers and practitioners to successfully provide NATAIS. Identifying barriers, improving evidence-based knowledge regarding their effectiveness, and developing guidelines are important pre-conditions to successfully implement interventions [38]. To the best of our knowledge, research on these pre-conditions regarding NATAIS is lacking and educational materials are not widely available.

Regarding the needs of the different stakeholders, cultural diversity is also a relevant element. Although a multicultural model was proposed to enhance multicultural considerations in animal-assisted interventions [60], there is a need to assess and evaluate NATAIS worldwide.

#### Financial resources

Another type of resource to be considered in the research agenda, is the costs related to implementing and maintaining NATAIS. Decision-making in mental healthcare should be based on the economic evaluations of interventions, that is “the comparative analysis of alternative courses of action in terms of both their costs and consequences” [61]. Economic evaluations in older adult care are scarce, which makes it difficult for researchers to make definitive statements about the cost-effectiveness of interventions compared to other interventions [62–64], especially with respect to NATAIS. Moreover, variation within the several NATAIS, e.g., animal- assisted interventions compared to nature-based interventions, limits the ability to generalize assumptions about their economic evaluation. The same applies to economic evaluation studies regarding loneliness [65]. Research on the cost-effectiveness of (NATAIS) interventions targeting loneliness in dementia is needed. In addition, the variations in outcome measurements make it difficult to estimate the effects of the applied interventions [65]. Therefore, to make strong statements about the cost-effectiveness of NATAIS used for reducing loneliness in PwND or for other purposes, economic evaluations are required.

### Activities

Activities encompass the actions needed to successfully complete a program [30], i.e., applying NATAIS to reduce the negative effects of social isolation and loneliness in PwND. These activities include research, the development of interventions, and training activities [30].

First, to improve evidence-based decision-making, it is important to translate the above described inputs into research activities. Although PwND may benefit from their participation in research, which is often experienced as pleasant, ethical considerations, methodological and practical issues make it challenging and time-consuming for researchers to involve PwND [66, 67]. Common examples of challenging issues include gaining ethical approval, collecting valid outcome measurements, and improving treatment adherence [66, 68]. It is important to consider these challenges and choose research methods carefully when designing studies for PwND. In addition to these issues in the context of PwND, other practical and ethical issues may arise when conducting research in NATAIS, e.g., ethical considerations regarding the involvement of specific animal species. This can limit the use of certain designs and under-represent several NATAIS. Despite the increased attention to animal welfare, more research is needed to ensure animal rights in research and interventions [69].

Second, to implement NATAIS successfully in the daily care and lives of PwND, their ecosystem, and healthcare settings, the proposed NATAIS should be feasible and practical to use. In general, and especially in times of increased social isolation, NATAIS should be accessible, easy to implement, and at low cost/profile. Other important facilitators for the implementation of psychosocial interventions to improve the quality of life in PwND are: awareness of the potential benefits, interventions in alignment with the current needs and stage of PwND, involvement of caregivers to support engagement, and interventions that may also support caregivers [70].

A third category of activities within the logic model involves investments in educational programs. Education improves the knowledge, skills, and attitudes of professional caregivers [71]. This is crucial for the early detection of social isolation and the prompt treatment of symptoms of loneliness. Moreover, there is a need to continuously educate and train professionals and gain broader public understanding of the benefits of NATAIS [72].

### Outputs

Outputs represent the products and services resulting from the activities [30]. Since outputs are important linkages between activities and outcomes, it is needed to constantly evaluate these outputs to ensure and improve quality of the delivered products and services [30]. Therefore, researchers should invest in conducting process evaluation studies on NATAIS. Data on process evaluation may help to gain insight into potential barriers and facilitators and help to fine-tune products, services and protocols on implementing certain interventions [30, 73]. In general, lack of time, information and training opportunities hinder successful implementation of interventions [38], and can be overcome by providing products and services as explained below.

#### Products (e.g., measurement tools, technological solutions, educational materials)

There is a need for valid measurement tools to assess social isolation and loneliness in PwND. It remains unclear whether cognitive decline and poor self-insight in PwND affects the validity and reliability of self-reports in PwND [74]. Therefore, research comparing and evaluating different ways of measuring social isolation and loneliness in PwND is needed. Besides a valid measurement on outcomes, other products may result from the different activities. One may think of technological solutions and educational materials, or tools to measure animal welfare. These tools may support professionals in conducting NATAIS successfully, and subsequently increase the well-being of PwND, their ecosystem and animal welfare.

#### Services (e.g., educational programs)

As discussed, for the successful implementation of interventions such as NATAIS, it is essential that both PwND and their caregivers (both family and professional) are aware of the benefits and risks associated with NATAIS. Although there are multiple organizations and universities focusing on human–animal interactions, there is a need to establish standards to competently and safely integrate animal-assisted interventions in practice, ensuring animal rights [72]. The same applies to other NATAIS.

### Outcomes

The initial focus of this project was on social isolation and loneliness in PwND, and their quality of life, especially during challenging times such as pandemics. However, the identified research themes might be seen as more general in nature, including other outcomes in NATAIS research. Although the effects found in previously published studies are promising for using NATAIS to achieve these outcomes in PwND, results often remain inconclusive and the available evidence remains limited [24, 25], which underscores the necessity of further research in this field.

In summary, the resulting research agenda embodies the identified research gaps in the field of NATAIS. Using a logic model as framework for developing the agenda, enabled us to structure the agenda, and ensured that the most important topics were discussed. Moreover, using a GCM procedure involving both practitioners and scientific experts, enabled to get input from stakeholders with various backgrounds, ensuring multiple perspectives. This diversity of input also facilitated group discussions and enabled to maintain focus. However, this study also has several limitations that should be considered. Although we intended to involve experts worldwide, all but one working group member were European, with most of them residing in the Netherlands. Moreover, although working group members were selected and chosen as representatives for older adults and (family) caregivers, we did not directly involve these target groups. This may limit the generalizability of the research questions.

## Conclusion

The primary objective of this project was to develop a research agenda aimed at stimulating research on NATAIS for PwND and their caregivers. The initial focus was on social isolation during pandemics. However, since our participants identified many open questions that did not relate exclusively to social isolation or pandemics, the focus was extended to the use of NATAIS for PwND in general. We developed a research agenda describing crucial research gaps and providing guidance for future research. It is highly important to address these issues in the future to ensure the best possible quality of life for PwND worldwide. To ensure success in such research, the collaboration between practitioners and researchers was identified as an important objective, essential for achieving better outcomes for PwND and their ecosystem, both in the short and long-term.

## Supplementary Information

Additional file 1.

Additional file 2.

## Data Availability

Data from the GCM procedure is available upon request. All statements that were retrieved from this procedure can be found in the research agenda, see Additional file 1.

## Abbreviations

GCM: Group concept mapping Procedure
PwND: People with neurodegenerative disease
NATAIS: Nature and animal-assisted intervention strategies
