# Correction: Developing a research agenda on NATure‑based and Animal‑assisted Intervention Strategies (NATAIS) in people with neurodegenerative diseases with a specific focus on social isolation and loneliness: a group concept mapping procedure

**DOI:** 10.1186/s12877-025-05835-7

**Published:** 2025-03-20

**Authors:** I. J. N. Declercq, R. Leontjevas, M. ‑J. Enders‑Slegers, M. Molog, D. L. Gerritsen, K. Hediger

**Affiliations:** 1 Department of Primary and Community Care, Radboud University Medical Centre, Nijmegen, The Netherlands; 2 Faculty of Psychology, Open University of the Netherlands, Heerlen, The Netherlands; 3 Faculty of Psychology, University of Basel, Basel, Switzerland

**Correction: BMC Geriatr 24, 795 (2024)**

10.1186/s12877-024-05387-2

Following publication of the original article [1], the authors reported inaccuracies regarding the generated citation of the article.

**Incorrect**:

I. J. N. Declercq^1,2*^, R. Leontjevas^1,2^, M.J. EndersSlegers^2^, M. Molog^2^, D. L. Gerritsen^1^, K. Hediger^2,3^ and on behalf of NATAIS Working Group (Sieka Bos, Simone de Bruin, Birgitta Erixon Halck, Sofie Hoorelbeke, Mayke Janssens, Yvonne van der Leest, Dorit van Meel, Zenithson Ng, Christine Olsen, Elizabeth Ormerod, Ingeborg Pedersen, Peter Reniers, Sandra Wesenberg, Jules Ellis, Jannes Eshuis, Nancy Gee, Richard Griffioen, Danielle Groenewoud, Sandra Haven-Pross, Sarah Janus, Daniel Mills, Victor Ojo, Patricia Pendry, Katharina Rosteius, Marjolein de Vugt and Sytse Zuidema)

**Correct**:

I. J. N. Declercq^1,2*^, R. Leontjevas^1,2^, M. J. EndersSlegers^2^, M. Molog^2^, D. L. Gerritsen^1^, and K. Hediger^2,3^

The original article [1] has been corrected.
