# Supplementary material for: Developing a research agenda on NATure-based and Animal-assisted Intervention Strategies (NATAIS) in people with neurodegenerative diseases with a specific focus on social isolation and loneliness: a group concept mapping procedure
Source: BMC Geriatr. 2024 Sep 28;24:795. doi: 10.1186/s12877-024-05387-2 (PMC11439302; doi:10.1186/s12877-024-05387-2)
Supplement: Supplementary file 1 — Additional file 1. [file 12877_2024_5387_MOESM1_ESM.pdf]

NATAIS

Research AGENDA

Nature-based and animal-assisted intervention strategies

A research agenda for people with neurodegenerative diseases, with a specific focus on social isolation

COLOPHON

Core team

Dr. Ruslan Leontjevas, Open University, Radboudumc, the Netherlands
Prof. dr. Karin Hediger, University of Basel, Basel, Switzerland
Prof. dr. Debby Gerritsen, Radboudumc, Nijmegen, the Netherlands
Ine Declercq, MSc, Vrije Universiteit Brussel, Belgium
Mascha Molog, MSc, Open University, the Netherlands
Prof. dr. Christel Moons, Ghent University, Belgium
Prof. em. dr. Marie-José Enders-Slegers, The International Association of Human-Animal Interaction Organizations

Team members (in alphabetical order)

Sieka Bos, MSc, Sieka Bos Coaching & Ondersteuning, the Netherlands
Dr. Simone de Bruin, Windesheim University of Applied Sciences, the Netherlands
Birgitta Erixon Halck, Sunnaas Rehabilitation Hospital, Dyrebar Omsorg, Norway
Sofie Hoorelbeke, Curando, Belgium
Dr. Mayke Janssens, Open University, Heerlen, the Netherlands
Yvonne van der Leest, Zorgerf Buiten-land en Buiten-Verblijf, the Netherlands
Dr. Dorit van Meel, ZHAW Life Sciences und Facility Management, Switzerland
Dr. Zenithson Ng, University of Tennessee College of Veterinary Medicine, USA
Dr. Christine Olsen, Norwegian Centre of Anthrozoology, Ekebyveien, Norway
Dr. Elizabeth Ormerod, The Society for Company Animals Studies, United Kingdom
Dr. Ingeborg Pedersen, Norwegian University of Life Sciences, Norway
Peter Reniers, MSc, Open University, Heerlen, the Netherlands
Prof. dr. Sandra Wesenberg, Alice-Salomon-University, Germany
Prof. dr. Jules Ellis, Open University, Heerlen, the Netherlands

Other contributors to the project (in alphabetical order)

Dr. Jannes Eshuis, dr. Nancy Gee, dr. Richard Griffioen, Danielle Groenewoud MSc, Sandra Haven-Pross MSc, dr. Sarah Janus, prof. dr. Daniel Mills, dr. Victor Ojo, prof. dr. Patricia Pendry, Katharina Rosteius MSc, prof. dr. Marjolein de Vugt, and prof. dr. Sytse Zuidema

NATAIS

CONTENT

Colophon		2
Introduction		3
Theme 0	Definitions and conceptualization	5
Theme 1	Challenges, risk factors, ethics, and animal welfare in NATAIS	7
Theme 2	Theoretical underpinnings and working mechanisms	9
Theme 3	Identifying target groups and matching NATAIS to their needs and preferences	11
Theme 4	Implementation of NATAIS	13
Theme 5	Methodological challenges	15
Theme 6	Research on accessible informal NATAIS	17
Theme 7	Caregivers: their attitudes, needs, and training and education	19
Theme 8	Technological solutions	21
Theme 9	Physical environment	23
Theme 10	Effects of different types of NATAIS in people with NDD	25

CITATION

NATAIS working group (2023). *NATure-based and Animal-assisted Intervention Strategies (NATAIS): A research agenda for people with neurodegenerative diseases, with a specific focus on social isolation.* EU Joint Programme – Neurodegenerative Disease Research.

Introduction

Keywords:
Research agenda, nature-based, animal-assisted, group concept mapping, neurodegenerative disease research

Authors:
Ruslan Leontjevas, Karin Hediger, Ine Declercq, Mascha Molog, Debby Gerritsen, Marie-José Enders-Slegers

Contact details:
roeslan.leontjevas@ou.nl

NATAIS
nature and animal assisted intervention strategies

WHAT IS THIS AGENDA ABOUT?

This research agenda focuses on nature-based and animal-assisted interventions (services) for people with neurodegenerative diseases and their formal and informal caregivers. It arose from the need to identify innovative and effective interventions that improve the quality of life during times of increased social isolation, such as the COVID-19 pandemic and its resultant social isolation

A research agenda acts as a catalyst for progress and collaboration, raises awareness within the research field and provides a sense of direction for academic and scientific activities.

Research consumers (e.g., practitioners, educators, and other professionals) can consult a research agenda to verify whether their roles and needs are addressed in future research activities. It is important to align the agenda's priorities with their efforts so that they can contribute to advancing knowledge and its practical applications.

Policy makers may use a research agenda to shape evidence-based policies and strategies. By referring to the agenda, policy makers can make well-informed decisions, such as those related to funding, which drive positive change, foster innovation, and improve the overall well-being of the target groups.

FUNDING

The Research Foundation – Flanders (FWO, Belgium) funded the development of the research agenda through the initiative of the EU Joint Programme – Neurodegenerative Disease Research (JPND). The working group’s activities were unpaid, with the exception of non-scientific administrative costs, travel expenses, and accommodation costs for work visits.

The EU Joint Programme – Neurodegenerative Disease Research (JPND) is the largest global research initiative aimed at tackling the challenge of neurodegenerative diseases. JPND aims to increase coordinated investment between participating countries in research aimed at finding causes, developing cures, and identifying appropriate ways to care for those with neurodegenerative diseases - www.jpnd.eu

NATAIS

This agenda introduces an umbrella term — NATure-based and Animal-assisted Intervention Strategies (NATAIS)—that subsumes all forms of strategies that use the potential benefits of interactions with nature and animals. The NATAIS project mirrors other concepts (see Theme 0) but is broader and considers the importance of meaningful unstructured activities with animals and connections with nature, independent of a formal program.

Nature-based activities and animal-assisted interventions have, among other benefits, been found to relieve pain (Han, et al., 2016); evoke positive emotions (Astell-Burt et al., 2022; Ellingsen-Dalskau et al., 2022; Olsen et al., 2016); partially reallocate attention externally (Mavrantza et al., 2023); decrease mental distress, and the use of doctor-prescribed depression medication (Chang et al., 2021; White et al., 2021); have a positive impact on mood and behavioral and psychological symptoms in dementia (Aarskog et al., 2019; Cheng et al., 2022), and have a positive impact on well-being in general (White et al., 2021).

Examples of NATAIS include nature-based activities, animal-assisted interventions, and green care farms. Different terms, such as canine-assisted therapy, wilderness therapy, horticultural therapy, and ecotherapy, are used to describe the involvement of nature and animals in a therapeutic context. While some activities may be conducted without a specific therapeutic goal, they can still have beneficial effects. As a result, the range of NATAIS is extensive, with all strategies sharing the common aim of harnessing the healing power of nature and animal interactions to support the well-being of individuals with a broad range of disorders, including neurodegenerative diseases.

Neurodegenerative diseases (NDD)

This research agenda adopts the definition of neurodegenerative diseases (NDD) provided by the EU Joint Programme – Neurodegenerative Disease Research (JPND; neurodegenerationresearch.eu).

BACKGROUND AND VISION

Neurodegenerative disease (NDD) can lead to various health issues. Roughly five out of six people with NDD often experience sleeping problems, feelings of anxiety and depression, and a lack of interest or motivation (Abraha et al, 2017) alongside the cognitive and physiological impairments.

There is a growing interest in exploring new ways to improve quality of life and reduce feelings of loneliness in people with NDD. The positive impact of spending time with nature and animals is currently drawing considerable attention. These types of activities or interventions can improve mood, relieve loneliness, and enhance well-being for people with NDD by providing a sense of meaning and a pleasurable experience.

This research agenda envisions a future where NATAIS benefit not only people with NDD, but also their caregivers, the animals and nature involved in the broader system of care. To that end, the focus is on both institutionalized long-term care and home care environments, ensuring the most comprehensive coverage of the care ecosystem.

This research agenda is committed to a One Health approach (Hediger et al., 2019), acknowledging the interconnection between people, animals, and their shared environment. This approach ensures that our research is ethical, balanced, and sustainable, and that it prioritizes the well-being of all involved.

AGENDA FORMAT

The development of the research agenda was structured around key elements of the logic model that were contextual factors, resources, activities, outputs, and outcomes. The Group Concept Mapping method (Rosas, 2017) was employed as a participatory approach to elicit input from team members (experts in nature-based interventions, animal-assisted interventions, and NDD). Initial research statements, generated through online brainstorming and subsequent sorting, were collected and thematically clustered. Common threads and overarching themes emerged (Trochim et al., 2017). Subsequently, these themes were used to build a logic model This procedural approach ensured a comprehensive and stakeholder-informed development of the research agenda, combining rigorous methodology with participatory engagement.

In total, the agenda comprises eleven themes (0 to 10) structured as follows: the title page, the work group members who restructured and (re-)formulated statements from the Group Concept Method procedure, definitions of concepts that are relevant within the theme, a list of the most obvious gaps that need to be addressed, and a list of research questions. Each theme includes a list of relevant publications. It is important to note that while these themes emerged from a robust procedure, they should not be viewed as exhaustive or immutable in future research.

NATAIS

LITERATURE SOURCES

- Abraha, I., Rimland, J. M., Trotta, F. M., Dell'Aquila, G., Cruz-Jentoft, A., Petrovic, M., ... & Cherubini, A. (2017). Systematic review of systematic reviews of non-pharmacological interventions to treat behavioural disturbances in older patients with dementia*BMJ Open,* 7(3), e012759.
- Aarskog, N. K., Hunskår, I., & Bruvik, F. (2019). Animal-assisted interventions with dogs and robotic animals for residents with dementia in nursing homes: A systematic review. *Physical & Occupational therapy in geriatrics*, *37*(2), 77-93.
- Astell-Burt, T., Hartig, T., Eckermann, S., Nieuwenhuijsen, M., McMunn, A., Frumkin, H., & Feng, X. (2022). More green, less lonely? A longitudinal cohort study. *International journal of epidemiology*, *51*(1), 99-110.
- Chang, SJ, Lee, J, An, H, et al. Animal-Assisted Therapy as an Intervention for Older Adults: A Systematic Review and Meta-Analysis to Guide Evidence-Based Practice. Worldviews on evidence-based nursing 2021;18(1):60-67.
- Chen, H., Wang, Y., Zhang, M., Wang, N., Li, Y., & Liu, Y. (2022). Effects of animal-assisted therapy on patients with dementia: A systematic review and meta-analysis of randomized controlled trials. *Psychiatry research*, *314*, 114619.
- Ellingsen-Dalskau, L. H., & Pedersen, I. (2022). Turning the ordinary into the extraordinary–Experiences of providing farm-based day care for people with dementia. *Wellbeing, Space and Society,* 3, 100119.
- Han, J. W., Choi, H., Jeon, Y. H., Yoon, C. H., Woo, J. M., & Kim, W. (2016). The effects of forest therapy on coping with chronic widespread pain: Physiological and psychological differences between participants in a forest therapy program and a control group. *International journal of environmental research and public health*, *13*(3), 255.
- Hediger, K., Meisser, A., & Zinsstag, J. (2019). A One Health Research Framework for Animal-Assisted Interventions. *International Journal of Environmental Research and Public Health,* 16(4), 640.
- Holwerda, T. J., Beekman, A. T., Deeg, D. J., Stek, M. L., van Tilburg, T. G., Visser, P. J., ... & Schoevers, R. A. (2012). Increased risk of mortality associated with social isolation in older men: only when feeling lonely? Results from the Amsterdam Study of the Elderly (AMSTEL). *Psychological Medicine,* 42(4), 843-853.
- Lai, N. M., Chang, S. M. W., Ng, S. S., Tan, S. L., Chaiyakunapruk, N., & Stanaway, F. (2019). Animal-assisted therapy for dementia. *Cochrane Database of Systematic Reviews*, 11).
- Numbers, K., & Brodaty, H. (2021). The effects of the COVID-19 pandemic on people with dementia. *Nature Reviews Neurology,* 17(2), 69-70.
- Olsen, C., Pedersen, I., Bergland, A., Enders-Slegers, M. J., Patil, G., & Ihlebæk, C. (2016). Effect of animal-assisted interventions on depression, agitation and quality of life in nursing home residents suffering from cognitive impairment or dementia: A cluster randomized controlled trial. *International Journal of Geriatric Psychiatry*, *31*(12), 1312-1321.
- Rosas, S. R. (2017). Group concept mapping methodology: Toward an epistemology of group conceptualization, complexity, and emergence. *Quality & Quantity*, *51*(3), 1403-1416.
- Trochim, W. M., & McLinden, D. (2017). Introduction to a special issue on concept mapping. *Evaluation and program planning*, 60, 166-75.
- White, M. P., Elliott, L. R., Grellier, J., Economou, T., Bell, S., Bratman, G. N., ... & Fleming, L. E. (2021). Associations between green/blue spaces and mental health across 18 countries. *Scientific reports*, *11*(1), 8903.

Theme 0
Definitions and conceptualization

Keywords:
Concepts, conceptual framework, definitions

Authors:
Ruslan Leontjevas, Debby Gerritsen, Marie-José Enders-Slegers, Ine Declercq, Mascha Molog, Dorit van Meel, Karin Hediger

Contact details:
karin.hediger@ou.nl

NATAIS
nature and animal assisted intervention strategies

WHAT IS THIS THEME ABOUT?

Input from stakeholders and target groups revealed the importance of clear definitions for NATAIS. Interdisciplinary research is necessary to provide a framework with existing or new terms for describing the concept of NATAIS.

BACKGROUND

Various terms exist for activities and therapies that involve green spaces (forests, parks, rooms with plants, etc.) and blue spaces (water basins, rivers, seas, oceans, etc.), and interactions with plants and animals. Often-used terms that may partially describe the concept of NATAIS are green care, nature-based medicine (activities, therapies), ecotherapy, animal-assisted activities and therapies. The NATAIS abbreviation may eventually be permanently adopted or replaced by another term. Establishing a universally accepted definition that incorporates both nature-based and animal-assisted intervention strategies across various settings is greatly needed.

DEFINITONS

Interdisciplinary conceptual research

Interdisciplinary conceptual research employs academic investigation that integrates concepts, theories, and methodologies from multiple scientific and professional fields. The integration of ideas and theories from different disciplines helps construct new, or enhance existing, frameworks, models, and approaches, further advancing knowledge in multiple areas.

Green care

Definitions of green care vary. Some definitions focus on formal care, while others include spontaneous unstructured interactions with nature that may not be professionally led. This inclusion of both formal and informal care and unstructured interactions overlaps with NATAIS used in this project. Formal care might encompass structured programs such as care farming, animal-assisted therapy sessions conducted by licensed therapists, or therapeutic horticulture programs led by trained facilitators.

Nature-based

Several terms start with ‘nature-based,’ including nature-based activities, nature-based therapy, and nature-based medicine. Like green care, these can be regarded as umbrella terms encompassing interventions that take place in natural spaces or which use both flora and fauna mainly outdoors; however, literature often focuses on the use of green or blue spaces and vegetation.

Ecotherapy

As with several other terms, definitions of ecotherapy vary across the literature. The term ecotherapy is often used to describe a regular, structured intervention conducted or supervised by trained professionals in a natural environment outdoors. Animal-assisted interventions can be provided as part of ecotherapy.

Animal-assisted interventions

Various sources related to human-animal interactions use the umbrella term animal-assisted interventions which include animal-assisted activities and animal-assisted therapies. Definitions and guidelines may vary, but the fundamental concepts often remain consistent: animal-assisted activities and services involve animals interacting with people for comfort and companionship, and animal-assisted therapies refer to structured interventions with specific therapeutic goals.

Concepts
Conceptual Framework
Definitions

CURRENT EVIDENCE AND RESEARCH GAPS

- Interdisciplinary research is essential for conceptual framing, allowing for comprehensive understanding and synthesis.
- Current research on related fields such as green care, nature-based interventions, and animal-assisted interventions is fragmented and lacks cohesion.
- Understanding how different definitions and terms impact the perception, implementation, and effectiveness of intervention strategies is needed.
- A universal definition encompassing all aspects of NATAIS is lacking. This may result in confusion and hinder effective communication and collaboration.

FUTURE RESEARCH QUESTIONS

- What are the underlying principles that unite and differentiate terms such as green care, nature-based medicine, and animal-assisted interventions?
- How can interdisciplinary research foster a comprehensive understanding of NATAIS?
- How can the various elements of NATAIS be integrated into a universally accepted definition?
- Are there cultural or regional variations in the understanding and implementation of NATAIS, and, if so, how can they be addressed?
- What methodologies and approaches are most suitable for conceptual research in NATAIS?
- How can interdisciplinary conceptual research contribute to a comprehensive understanding of the role of nature-based and animal-assisted interventions in mitigating the negative impacts of social isolation and loneliness during global pandemics such as COVID-19?

LITERATURE SOURCES

- Beers, P. J., & Bots, P. W. (2009). Eliciting conceptual models to support interdisciplinary research. Journal of Information Science, 35(3), 259-278.
- Berget, B., Braastad, B., Burls, A., Elings, M., Hadden, Y., Haigh, R., ... & Haubenhofer, D. K. (2010). Green Care: a Conceptual Framework. A Report of the Working Group on the Health Benefits of Green Care (No. 866). Loughborough University.
- Bodnar, S. (2023). Introduction to the Special Issue: A Collaborative Definition of Ecotherapy. Ecopsychology, 15(3), 206-213.
- La Puma, J. (2023). What is nature-based medicine and what does it do? American Journal of Lifestyle Medicine, 15598276221148395.
- Kruger, K. A., & Serpell, J. A. (2010). Animal-assisted interventions in mental health: Definitions and theoretical foundations. In Handbook on animal-assisted therapy (pp. 33-48). Academic Press.
- Machado, A., & Silva, F. J. (2007). Toward a richer view of the scientific method. The role of conceptual analysis. The American psychologist, 62(7), 671–681.
- Mendes, L., Oliveira, J., Barbosa, F., & Castelo-Branco, M. (2022). A conceptual view of cognitive intervention in older adults with and without cognitive decline—A systemic review. Frontiers in Aging, 3, 844725.

NATAIS

Theme 1
Challenges, risk factors, ethics, and animal welfare in NATAIS

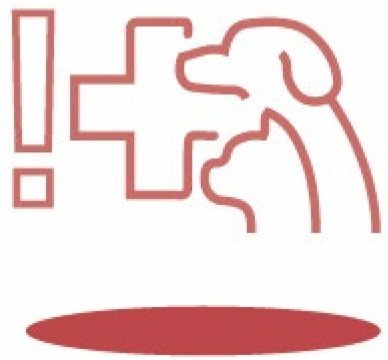

Keywords:
Challenges, risk factors, ethics, animal welfare, One Health, One Welfare, animal supported services, nature

Authors:
Marie-José Enders-Slegers, Zenithson Ng, Christel Moons, Elisabeth Ormerod, Christine Olsen

Contact details:
Marie-Jose.Enders@ou.nl

NATAIS
nature and animal assisted intervention strategies

WHAT IS THIS THEME ABOUT?

This theme addresses the challenges, risk factors, and ethical issues related to the animals and nature involved in NATAIS for people with NDD. When research focuses solely on the effects of NATAIS on the well-being of people with NDD, other important considerations such as animal welfare and respectful use of nature might be overlooked. In this theme, we explore the difficulty of finding a balance between human benefit and nature/animal burden in NATAIS from the perspective of a One Health/One Welfare principle.

BACKGROUND

Growing evidence of the beneficial effects of NATAIS for people with NDD, their families, and professional caregivers makes these interventions popular; however, challenges, risk factors, ethics, and the welfare of animals are often overlooked. Attention to challenges and risk factors is needed to ensure that interventions take place in an ethical way and do not jeopardize the well-being of people, the welfare of animals, or the health of nature.

Guidelines and protocols that safeguard the well-being of humans, animals, and the natural environment in NATAIS also need to be developed. Education about NATAIS should be provided in many disciplines, as well as for formal and informal caregivers of people with NDD.

Challenges regarding the involvement of animals and nature in NATAIS include aspects such as attention to potential negative effects of NATAIS on clients, animals, and nature; using validated instruments for measuring animal welfare in the short and long term; safety risks (e.g., falling, eating) for people with NDD in natural environments.

Furthermore, risk factors need to be considered such as mismatch between animal, client, environment, and intervention; potential spread of diseases; over burdening of the animals; zoonotic transmissions; injuries; unrealistic expectations of what animals can provide.

DEFINITONS

Ethics

The One Health/One Welfare approach emphasizes that the welfare of humans, animals, and nature is interconnected. Since humans, animals, and nature are involved in NATAIS, the welfare of each needs to be considered of equal importance and guaranteed to ensure ethical NATAIS.

Animal welfare

Animal welfare can be defined as the physical and mental state of an animal in relation to the conditions in which it lives and dies. The terms “animal welfare” and “animal well-being” are often used interchangeably, though the term well-being is sometimes considered less precise and most scientists instead use the term welfare. The term quality of life is often used in the context of companion animals. Both the quality of life and the welfare of an animal can be described variously (e.g., good, poor, etc.); however, whereas welfare can be used to describe a very short-term situation, quality of life is usually applied to situations involving (longer) spans of time of at least a few days.

One Health/One Welfare

One Health refers to the concept that the health of people is connected to the health of animals and our shared environment (nature). The One Health strategy encourages multidisciplinary collaboration and communication in relation to all aspects of interactions with the environment and health care for humans and non-human animals.
The One Welfare approach emphasizes that the concept of welfare is identical whether applied to humans or to non-human animals and should be incorporated into teaching about both.

Challenges
Risk Factors
Ethics
Animal Welfare
One Health / One Welfare
Animal supported services
Nature

CURRENT EVIDENCE AND RESEARCH GAPS

- Interdisciplinary research involving ethologists, veterinarians, and practitioners is essential for finding solid methods of measuring animal welfare in therapy dogs, equines, and other animals involved in NATAIS.
- Based on the literature on animal welfare, there is no evidence to demonstrate that animal-assisted interventions result in significant distress in appropriately selected and worked animals.
- There is a lack of information on studies regarding the welfare of farm animals used for health care; however, it has been suggested that involvement in a care farming may not necessarily be beneficial for animals.
- There is a lack of information on ethics regarding NATAIS during the COVID-19 pandemic.

FUTURE RESEARCH QUESTIONS

- What are the effects of dose (frequency, duration, and intensity) and other characteristics of therapeutic sessions on welfare parameters during NATAIS with animals?
- How do the welfare outcomes for an animal differ when it operates in an unfamiliar environment to which it has been transported, as compared to when it is in its own home?
- What are the ethical considerations involved in giving equal priority to human and animal well-being in NATAIS research and practice?
- What knowledge do various health and social care professionals possess about the potential risks for humans and animals associated with NATAIS, and do they employ a multidisciplinary approach?
- Are health and social care professionals implementing appropriate protocols to help ensure the welfare and safety of all participants in NATAIS—within both care facilities and in-home care? Are they aware of existing protocols and best practice guidelines?
- To what extent are undergraduates of the health, social care, and veterinary professions taught about nature-based approaches and human-animal interactions, including ethical aspects, evidence-based health, and social benefits?
- How can challenges, risk factors, ethics, and animal welfare in NATAIS be taught in the public health curriculum of medical and veterinary students, including risks of injury and zoonoses, especially for people with NDD?
- How can the principles of One Health/One Welfare be effectively applied to address the challenges, risk factors, and ethical considerations surrounding the involvement of animals and nature in NATAIS for individuals with NDD during times of heightened social isolation, thereby ensuring that the well-being of individuals, animals, and the environment is harmoniously balanced and preserved?

NATAIS

LITERATURE SOURCES

- Boroujeni, A. (2022). Horticultural therapy in improving fine motor, social and communication skills: A study from the Gharaunda Centre of Jankalyan Divyang Punarwasan Kendra in Latur, Maharashtra. *Journal of Educational Research and Policies*, 4 (7).
- Bowler, D. E., Buyung-Ali, L. M., Knight, T. M., & Pullin A. S. (2010). A systematic review of evidence for the added benefits to health of exposure to natural environments. *BMC Public Health*, 10(1), 1-10.
- Broom, D. (2006). Behaviour and welfare in relation to pathology. *Applied Animal Behaviour Science*, 97, 73-83.
- Chang, S. J., Lee, J., An, H., Hong, W. H., & Lee, J. Y. (2021). Animal-assisted therapy as an intervention for older adults: A systematic review and meta-analysis to guide evidence-based practice. *Worldviews on Evidence-Based Nursing*, 18(1), 60-67. Epub 2020 Dec 5. PMID: 33277977.
- Chun, M. H., Chang, M. C., & Lee S. J. (2017) The effects of forest therapy on depression and anxiety in patients with chronic stroke, *International Journal of Neuroscience*, 127(3), 199-203.
- Gorman, R. (2017). Therapeutic landscapes and non-human animals: The roles and contested positions of animals with care farming assemblages. *Social & Cultural Geography*, 18, 315–335. 2. IAHAIO White Paper www.iahaio.org; SCAS Code of Practice in AAI.
- Knight, A., Phillips, C., & Sparks, P. (Eds.). (2022). Routledge Handbook of Animal Welfare (1st ed.). Routledge.
- Mavrantza, A. M., Bigliassi, M., & Calogiuri, G. (2023). Psychophysiological mechanisms underlying the effects of outdoor green and virtual green exercise during self-paced walking. *International Journal of Psychophysiology*, 184. 39-50.
- Moberg, G. P. (2000). Biological response to stress: Implications for animal welfare. In: Moberg, G.P. and Mench, J.A., (Eds.). The Biology of Animal Stress: Basic Principles and Implications for Animal Welfare, CABI Publishing, New York, 1-21.
- Odendaal, J.S. (2000). Animal-assisted therapy—magic or medicine? *Journal of Psychosomatic Research*, 49(4), 275-280.

Theme 2

Theoretical underpinnings and working mechanisms

Keywords:
Theory, mechanism, effective components

Authors:
Mayke Janssens, Mascha Molog, Dorit van Meel, Simone de Bruin, Birgitta Erixon Halck

Contact details:
mayke.janssens@ou.nl

NATAIS
nature and animal assisted intervention strategies

WHAT IS THIS THEME ABOUT?

The focus of this theme is on exploring the theoretical foundations of NATAIS and disentangling their effective components and working mechanisms. Insight into the underlying mechanisms is needed to develop new, or refine existing, intervention strategies. It is important to ascertain whether such mechanisms remain effective during periods of increased social isolation. A coherent theoretical framework can help to predict outcomes of new interventions and existing interventions in under-researched target groups and in challenging times, such as during the COVID-19 pandemic.

BACKGROUND

The field of NATAIS research can benefit from solid explanatory theories that help in understanding the working mechanisms. This is especially the case for the fundamental question of what makes NATAIS distinct from other types of (psychosocial) interventions. For instance, considering the effects of NATAIS in green care farms as described in another theme, individuals with NDD may perceive themselves as employees or volunteers rather than patients, given that the care farm environment more closely resembles a non-institutional setting; however, other explanations inherent specifically to greenery or animal interactions are also possible.

DEFINITONS

Scientific theory

A scientific theory is a well-substantiated explanation of an aspect of the natural world, in this case the effectiveness of NATAIS, based on extensive and repeated experimentation. A theory explains known facts and allows scientists to make predictions based on what they should observe if the theory holds true. Therefore, a scientific theory of NATAIS should explain why and how NATAIS works. A good theory should remain valid during routine times, as well as during crisis situations such as global pandemics.

Mechanism

A mechanism is a plausible account of the process that establishes a systematic relationship between variables. Mechanisms refer to the processes necessary to yield a certain outcome. Mechanisms can be represented in a model (a simplified representation of reality) that provides a means of calculation or simulation.

Framework

A framework is not as specific as a model but outlines a broad view of a phenomenon. By identifying the key factors, variables, or concepts and their presumed relationships, frameworks provide a structure or set of guidelines for understanding a complex issue.

Theory
Mechanism
Effective Components

CURRENT EVIDENCE AND RESEARCH GAPS

- Several theories have been proposed that may explain the effects of NATAIS or their elements. Examples include stress reduction theory, attention restoration theory, social support theory, and attachment theory.
- Several mechanisms may explain NATAIS effects. Examples are social interaction or support, reinforcement of identity, meaningful engagement, positive risk-taking, sense of empowerment, distraction, motivation, physical activity, and activation of the oxytocin system. Since the effects of NATAIS can be manifold, it is reasonable to assume that there are probably several mechanisms at work.
- However, it is not clear which mechanisms are (especially) relevant in the context of NDD and how these potentially affect one another.
- Currently, there is no comprehensive theoretical framework that could explain the potential positive effects of NATAIS for people with NDD.
- It is not clear whether mechanisms of NATAIS change during pandemics such as COVID-19.

FUTURE RESEARCH QUESTIONS

- What are the elements of NATAIS that explain effects in people with NDD?
- What are the mechanisms behind (elements of) NATAIS in people with NDD?
- Are there mechanisms for NATAIS that are specific to NDD?
- What is the relationship between multiple mechanisms of NATAIS in people with NDD?
- What theoretical framework(s) can be applied to NATAIS in NDD?
- How do mechanisms of NATAIS fit within this potential theoretical framework and how do these differ for people with different types of NDD?
- Is there a different effect when people with NDD interact with an animal compared to when they interact with a human?
- What are the implications of global pandemics such as COVID-19 for theoretical underpinning and specific elements/mechanisms of NATAIS? How might these mechanisms adapt or evolve during such periods of heightened social isolation?
- Future research should be aimed at developing a comprehensive theoretical framework regarding the use of NATAIS in NDD during routine times and during crisis situations like pandemics. The accompanying mechanisms should be investigated, as well as how these mechanisms relate to each other and whether these are specific for NDD.

LITERATURE SOURCES

- Beetz, A. M. (2017). Theories and possible processes of action in animal assisted interventions, *Applied Developmental Science*, 21(2), 139-149.
- Hartig,T., Mitchell, R., de Vries, S., & Frumkin, H. (2014). *Nature and health*. *Annual Review of Public Health*, 35, 207-228.
- Kaplan, S. (1995). The restorative benefits of nature: Toward an integrative framework. *Journal of environmental psychology*, 15(3), 169–182.
- Mmako, N. J., Courtney-Pratt, H., & Marsh, P. (2020). Green spaces, dementia and a meaningful life in the community: A mixed studies review. *Health & Place*, *63*, 102344.
- Murroni, V., Cavalli, R., Basso, A., Borella, E., Meneghetti, C., Melendugno, A., & Pazzaglia, F. (2021). Effectiveness of therapeutic gardens for people with dementia: A systematic review. *International journal of environmental research and public health*, *18*(18), 9595.
- Serpell, J., McCune, S., Gee, N., & Griffin, J. A., (2017). Current challenges to research on animal-assisted interventions. *Applied Developmental Science*, 21(3), 223-233.
- Ulrich R. S., Simons, R. F., Losito, B. D., Fiorito, E., Miles, M. A., & Zelson, M. (1991). Stress recovery during exposure to natural and urban environments. *Journal of environmental psychology*, 11(3), 201–230.
- Wilson, E. O. (1984). *Biophilia*. Cambridge, MA: Harvard University Press.

NATAIS

Theme 3
Identifying target groups and matching their needs and preferences to NATAIS

Keywords:
Characteristics, age, gender, ethnicity, background, individual needs, preferences, target groups

Authors:
Dorit van Meel, Simone de Bruin, Birgitta Erixon Halck, Mayke Janssens, Mascha Molog

Contact details:
sr.de.bruin@windesheim.nl; dorit.vanmeel@zhaw.ch

NATAIS
nature and animal assisted intervention strategies

WHAT IS THIS THEME ABOUT?

This theme is about the characteristics, needs, and preferences of different groups of people with NDD that may receive or conduct NATAIS. This theme addresses the possibilities of matching NATAIS to the needs and preferences of people with NDD.

BACKGROUND

Recognizing and defining target groups is important for developing interventions, providing tailored services, and allocating resources effectively. By identifying specific target groups, organizations and service providers can better understand the unique needs, challenges, and preferences of the individuals they aim to support.

Literature addressing NATAIS target groups is limited. Most studies primarily focus on people with dementia, while little information is available about individuals with NDD who are not experiencing cognitive problems. Moderating effects need to be explored regarding needs and preferences, and individual characteristics (e.g., diagnosis, age, gender, ethnicity, and cultural background).

For understanding cultural influences and the generalizability of the results, there is a lack of worldwide evaluation for certain NATAIS. For example, horticultural therapy has mainly been studied in Asia, while care farming has predominantly been studied in Europe. Consequently, unanswered questions remain about which NATAIS are effective for whom and why.

DEFINITONS

Target groups

Individuals can share characteristics or attributes that reflect demographics, health conditions, or disabilities. Some characteristics or attributes such as an individual's age or the nature of an NDD may be unchangeable. As previous research has shown, other personal characteristics, such as attitudes or skills, may be more flexible and still influence outcomes. Therefore, these should be accounted for in interventions.

Needs

The needs of individuals with NDD are often determined by the specific disease and their personal characteristics. For example, a person may require support and training to improve social skills, enhance communication abilities, and foster meaningful relationships with others in times of cognitive or physical deterioration. The level of support required for daily living will depend on the severity of the individual's health condition.

Preferences

Individuals may have their own unique preferences about things like hobbies and interests, social interactions, sensory experiences, and environmental conditions, etc. Understanding and respecting these preferences is crucial in providing person-centered care and support. Some preferences may remain the same over a long period of time while others may evolve or be influenced by personal growth and experiences.

Characteristics
Background
Individual Needs
Preferences
Target Groups

CURRENT EVIDENCE AND RESEARCH GAPS

- Most studies on NATAIS focus on older people in general or on people with Alzheimer's disease/dementia. More insight into the effects for individuals with other types of NDD is needed.
- Most NATAIS studies investigate horticultural therapy/therapeutic horticulture, AAI, and care farming, while fewer studies address other types of NATAIS, such as forest therapy.
- Most studies examine specific settings/symptoms and methods. Overview studies that investigate multi-factor relationships in real-life settings are lacking.
- Studies that investigate the combined effects of animals, plants, and natural settings on people with NDD are lacking. For example, available evidence indicates that:
    - Generally, care farms with day services serve younger people or individuals with mild to moderate dementia, while those in the later stages of the disease are offered 24-hour nursing care.
    - Care farms can be attractive for individuals both with and without an agricultural background. It can be hypothesized that, similarly, animal-assisted interventions can be attractive for those both with and without previous interactions with animals.
- Information on how cultural backgrounds may influence the implementation and effects of NATAIS is currently limited.
- Limited research indicates that a global health care crisis such as the COVID-19 pandemic can have different impacts on participant interactions with specific animal species or plants. Participants with different profiles can be affected differently.

FUTURE RESEARCH QUESTIONS

- What are the characteristics of the different groups of people with NDD currently using NATAIS?
- What are the needs and preferences of different groups of people with NDD in relation to NATAIS?
- What kinds of NATAIS (e.g., AAI, care farming, horticulture, urban greenery, wild greenery, greenery, forest therapy) and/or what specific elements of these strategies (e.g., activities with plants, activities with animals, views of nature, social interactions, etc.) are preferred by whom (e.g., diagnosis, age, gender, ethnicity, cultural background, life experiences)?
- To what extent are there any differences regarding the needs, preferences, and benefits of different groups regarding NATAIS (e.g., cultural background, ethnicity, SES, gender, religion)?
- What are the reasons behind different effects of similar NATAIS on different people? And how can these findings be used to optimize nature-based program options?
- To what extent are existing NATAIS aligned with the capacities, needs, and preferences of people with NDD (and their caregivers), considering dementia stage, dementia type, ethnicity, cultural background, etc.?
- How can NATAIS be adapted to cater to the distinctive characteristics, needs, and preferences of diverse groups of individuals with NDD, thereby ensuring targeted support and enhanced well-being during periods of heightened social isolation caused by global pandemics such as COVID-19?

LITERATURE SOURCES

- Chen, H., Wang, Y., Zhang, M., Wang, N., Li, Y., & Liu, Y. (2022). Effects of animal-assisted therapy on patients with dementia: A systematic review and meta-analysis of randomized controlled trials. *Psychiatry Research*, 314.
- De Bruin, S. R., Pedersen, I., Eriksen, S., Hassink, J., Vaandrager, L., & Patil, G. G. (2020). Care farming for people with dementia; what can healthcare leaders learn from this innovative care concept? *Journal of Healthcare Leadership,* 12, 11–18.
- Garcia-Llorente, M., Rubio-Olivar, R., & Gutierrez-Briceno, I. (2018). Farming for life quality and sustainability: A literature review of green care research trends in Europe. *International journal of environmental research and public health*, 15(6).
- Heród, A., Szewczyk-Taranek, B., & Pawłowska, B. (2022). Therapeutic horticulture as a potential tool of preventive geriatric medicine improving health, wellbeing and life quality – A systematic review. *Folia Horticulturae,* 34(1), 85-104.
- Kimicki, M. L., Edwards, N. E., Richards, E., & Beck, A. M. (2019). Animal-assisted intervention and dementia: A systematic review. *Clinical nursing research*, 28(1), 9-29.
- Orr, N., Abbott, R., Bethel, A., Paviour, S., Whear, R., Garside, R., & Coon, J. T. (2023). What are the effects of animals on the health and wellbeing of residents in care homes? A systematic review of the qualitative and quantitative evidence. *BMC geriatrics*, 23(1), 170.
- Otman, R., Johari, N. N., Hatta, F. A. M., Ramya, R., Sulaiman, W. S. H. W., & Latif, N. H. M. (2022). Forest therapy and design preferences for senior citizen's healthcare enhancement at Health Forest, Terengganu. *New Design Ideas,* 6(1), S. 101-125.
- Shoesmith, E., Surr, C., & Ratschen, E. (2023). Animal-assisted and robotic animal-assisted interventions within dementia care: A systematic review. *Dementia*, 22(3), 664-693.
- Vimal, R. (2022). The impact of the Covid-19 lockdown on the human experience of nature. *Science of the Total Environment*, *803*, 149571.
- Wang, Z., Zhang, Y., Lu, S., Tan, L., Guo, W., Lown, M., ... & Liu, J. (2022). Horticultural therapy for general health in the older adults: A systematic review and meta-analysis. *PLoS one,* 17(2). e0263598.

NATAIS

Theme 4
Implementation of NATAIS

Keywords:
Implementation, application, operationalization, putting into practice, diffusion, distribution, barriers, facilitators, evaluation

Authors:
Debby Gerritsen, Ingeborg Pedersen, Sofie Hoorelbeke, Yvonne van der Leest, Peter Reniers

Contact details:
debby.gerritsen@radboudumc.nl

NATAIS
nature and animal assisted intervention strategies

WHAT IS THIS THEME ABOUT?

This theme focuses on the practical implementation of NATAIS, encompassing implementation strategies in both intramural and extramural settings. To effectively implement NATAIS, it is crucial to acquire insights into the barriers and facilitators of conducting NATAIS within various types of NDD and across different contexts, all while considering the diverse forms of NATAIS.

BACKGROUND

To effectively implement NATAIS in residential settings and within the community, a comprehensive understanding of both successful and unsuccessful implementation strategies is essential, along with insights into the barriers and facilitators of implementation. NATAIS interventions often adopt a perspective of social prescribing, yet there is a scarcity of research exploring NATAIS implementation within this approach, as well as in formal treatment contexts such as therapy.

The understanding of NATAIS would be greatly enhanced by incorporating process evaluations into effectiveness studies. Achieving successful implementation requires consideration of factors such as financial resources, sociocultural elements, legal considerations, and the attitudes towards NATAIS held by people with NDD, their families, and professional caregivers. Various theoretical frameworks, such as the Theory of Planned Behavior, can be employed to develop robust implementation plans. The presence of economic evaluations demonstrating the cost-effectiveness of NATAIS, as well as a thorough understanding of legal intricacies, are pivotal prerequisites for the implementation of NATAIS. These insights can further aid policymakers in selecting optimal strategies for further promotion and funding.

DEFINITONS

Implementation

Implementation involves translating ideas, strategies, and policies into actions, activities, and structures within a specific context or setting. Implementation typically encompasses tasks such as organizing resources, executing planned activities, managing stakeholders, and monitoring progress to ensure that the intended goals and outcomes are achieved effectively and efficiently.

Social prescribing

Social prescribing involves referring clients to non-clinical settings with the goal of addressing their social needs. This approach can contribute to enhancing clients' societal connection and improve their mental health. Examples of social prescribing encompass activities such as community gardening or becoming a member of a walking club.

Economic evaluation

An economic evaluation involves comparing the costs and benefits of various alternative interventions. Various forms of economic evaluation exist, including cost-effectiveness analysis, cost-benefit analysis, and cost-consequence analysis; however, a gap exists in the economic evaluation of NATAIS, especially within the realm of community aged care.

Implementation
Application
Operationalization
Putting into Practice
Diffusion
Distribution
Barriers
Facilitators
Evaluation

CURRENT EVIDENCE AND RESEARCH GAPS

- Community-dwelling people with NDD who establish connections within society, receive support, and engage in NATAIS interactions in public spaces, tend to experience a higher quality of life.
- Conducting an economic evaluation that considers family care services and outcome measures related to NATAIS in care is necessary, potentially facilitating the implementation of NATAIS.
- To ensure the delivery of high-quality care through NATAIS, a fundamental shift in the social culture of both residential and home care settings is essential, embracing the principles of NATAIS.
- Nature-based social prescribing approaches, such as community gardening, have the potential to promote NATAIS and foster the integration and the acceptance of people with NDD within the community.
- Utilizing nature-based social prescribing, such as engaging in community gardening, could potentially foster NATAIS and enhance the acceptance of residents with NDD within the community.
- Community-dwelling people with NDD who establish connections within society, receive support, and engage in NATAIS interactions in public spaces can significantly contribute to the creation of dementia-friendly neighborhoods.
- Nature-based social prescribing was impeded by changed conditions during the COVID-19 pandemic, such as lockdowns, and social distancing, which increased social isolation in vulnerable groups, such as older adults (with NDD).

FUTURE RESEARCH QUESTIONS

- What are the costs associated with implementing, applying, and sustaining NATAIS in care settings, compared to other interventions?
- How can the caregiving culture be transformed to facilitate the acceptance of NATAIS as a standard form of care?
- What is the impact of implementing social prescribing through NATAIS on the integration and acceptance of people with NDD within the community?
- What legal and ethical considerations are either required or obstructive for the implementation and integration of NATAIS within the community?
- In what ways can NATAIS contribute to the realization of dementia-friendly neighborhoods?
- How can the implementation of NATAIS be strategically planned and adapted for both intramural and extramural settings, while considering unique challenges presented by global pandemics such as COVID-19 and the ramifications of prolonged social isolation for individuals with NDD?

NATAIS

LITERATURE SOURCES

- Bulamu, N. B., Kaambwa, B., & Ratcliffe, J. (2018). Economic evaluations in community aged care: A systematic review. *BMC Health Services Research,* 18(1), 967.
- Fixsen A., & Barrett, S. (2022). Challenges and approaches to green social prescribing during and in the aftermath of COVID-19: A qualitative Study. *Frontiers in Psychology*, 13, 861107.
- Leavell, M. A., Leiferman, J. A., Gascon, M., Braddick, F., Gonzalez, J. C., & Litt, J. S. (2019). Nature-based social prescribing in urban settings to improve social connectedness and mental well-being: A review. *Current Environmental Health Reports*, 6(4), 297-308.
- Podgorica, N., Flatscher-Thöni, M., Deufert, D., Siebert, U., & Ganner, M. (2021). A systematic review of ethical and legal issues in elder care. *Nursing Ethics*, 28(6), 895-910.
- Shier, V., Khodyakov, D., Cohen, L. W., Zimmerman, & S., Saliba, D. (2014). What does the evidence really say about culture change in nursing homes? *The Gerontologist,* 54(Suppl_1), S6-S16.
- Sturge, J., Nordin, S., Sussana Patil, D., Jones, A., Légaré, F., Elf, M., & Meijering, L. (2021). Features of the social and built environment that contribute to the well-being of people with dementia who live at home: A scoping review. *Health & Place*, 67, 102483.
- Wieczorek, E., Kocot, E., Evers, S., Sowada, C., & Pavlova, M. (2022). Key care provision aspects that affect care transition in the long-term care systems: Preliminary review findings. *International Journal of Environmental Research and Public Health,* 19(11), 6402.

Theme 5
Methodological challenges

Keywords:
Measurements, designs, qualitative research, quantitative research, mixed methods, reviews

Authors:
Ruslan Leontjevas, Ine Declercq, Sieka Bos, Jules Ellis, Karin Hediger

Contact details:
roeslan.leontjevas@ou.nl

NATAIS
nature and animal assisted intervention strategies

WHAT IS THIS THEME ABOUT?

Applying a thorough research methodology during pandemics and other crisis situations is challenging. It is important to select the best methods that align with scientific research questions and are applicable to the specific population and situation.

BACKGROUND

Scientists collect and analyze information to draw conclusions. Their research methods vary according to specific research questions, target populations, and situations. For example, randomized controlled trials are considered the gold standard for questions on the effectiveness of interventions; however, it is not always possible or ethical to vary conditions in a NATAIS trial. This implies that NATAIS researchers should be continuously updated on methodological innovations and actively encouraged to conduct research on methodological issues as well. Developments in artificial intelligence and other innovations need to be closely monitored to inform researchers on the best ways to conduct research.

DEFINITONS

Design

Research design serves as a strategic framework for collecting and analyzing qualitative or quantitative information, and enabling researchers to answer scientific questions. For target groups of interest, such as people with NDD or their family and professional caregivers, research design determines how participants are assigned to either intervention or control groups. Data can be collected at a single point or at multiple time points.

Conceptualization and operationalization

For collecting and analyzing information, research ideas need to be converted into concrete definitions. Both researchers and research consumers use definitions or concepts to understand research ideas and research outcomes.

After the conceptualization process, ideas and outcomes need to be translated into specific variables or indicators that can be observed and quantified. This process is known as operationalization, which involves measuring variables using instruments such as questionnaires and observations.

Validity and reliability

Good research is based on valid and reliable information. A study’s internal validity reflects the certainty that study results represent the real world regarding the definitions and correlational or cause-and-effect relationships. External validity represents the degree of confidence that the results are not only applicable to the study but also to other situations, groups of people, and events. Instruments are valid when they measure what they are intended to measure. For this, instruments should also be reliable. This means that instruments provide the same results when they measure an unchanged construct at different time points, or when instruments are used by different people or in different situations.

Measurements
Designs
Qualitative Research
Quantitative Research
Mixed Methods
Reviews

CURRENT EVIDENCE AND RESEARCH GAPS

- The number of studies on NATAIS in the general population and in people with NDD is growing; however, practical issues, costs, and ethical considerations can limit the use of certain designs. Therefore, some NATAIS might be under-represented in research (e.g., studies that compare different animal species).
- Severe cognitive problems and poor self-insight may result in invalid or unreliable self-reports in some people with NDD.
- During pandemics and enforced social isolation, some NATAIS can still be used, but knowledge on how to deliver NATAIS might be limited among stakeholders, including caregivers.
- Process evaluation studies on NATAIS remain limited. Process evaluations are needed to provide insight into the internal and external validity of specific interventions.

FUTURE RESEARCH QUESTIONS

- Which research designs can be used when participants cannot be randomized into different conditions (e.g., with or without a pandemic) due to practical or ethical reasons?
- Which methods of data collection (i.e., observations, self-report, and proxy-based questionnaires) are valid and reliable for NATAIS research in people with NDD?
- Which instruments can be used in NATAIS research with stakeholders that cannot provide valid and reliable information on their own (e.g., how to gather information in people with severe cognitive problems and in animals involved in AAI)?
- What is the best way to measure variables of interest in people with NDD, or important others, in research on NATAIS during pandemics or increased social isolation?
- Which alternatives to randomized controlled trials can be used when conditions with and without NATAIS elements cannot be altered or modified in times of pandemics or social isolation?
- Which frameworks for process evaluation can be used for understanding the internal and external validity of NATAIS in general and in studies with stakeholders who cannot provide valid and reliable data?
- What is the most appropriate and ethically sound research methodology that can be effectively applied during pandemics or crisis situations to investigate the efficacy of NATAIS, considering the alignment of methodological choices with scientific research questions, the unique characteristics of the target population, and the exigencies of the crisis context?

LITERATURE SOURCES

- Bucholc, M., James, C., Khleifat, A. A., Badhwar, A., Clarke, N., Dehsarvi, A., ... & Ranson, J. M. (2023). Artificial Intelligence for Dementia Research Methods Optimization. *arXiv preprint arXiv:2303.01949*.
- Griffiths, A. W., Smith, S. J., Martin, A., Meads, D., Kelley, R., & Surr, C. A. (2020). Exploring self-report and proxy-report quality-of-life measures for people living with dementia in care homes. *Quality of Life Research*, *29*, 463-472.
- Phenwan, T., Sixsmith, J., McSwiggan, L., & Buchanan, D. (2021). A rapid review of internet mediated research methods with people with dementia: Practical, technical and ethical considerations. *The Qualitative Report*, *26*(11), 3318-3341.
- Shannon, K., Montayre, J., & Neville, S. (2021). Nothing about us without us: research methods enabling participation for aged care residents who have dementia. *International Journal of Qualitative Methods*, *20*, 16094069211055938.
- Taylor, E. M., Robertson, N., Lightfoot, C. J., Smith, A. C., & Jones, C. R. (2022). Nature-based interventions for psychological wellbeing in long-term conditions: A systematic review. *International Journal of Environmental Research and Public Health*, *19*(6), 3214.

NATAIS

Theme 6
Research on accessible informal NATAIS

Keywords: Informal strategies, environmental stimuli, working environment, purposeful activities, accessible interventions

Authors:
Peter Reniers, Ingeborg Pedersen, Sofie Hoorelbeke, Yvonne van der Leest, Debby Gerritsen

Contact details:
peter.reniers@ou.nl

NATAIS
nature and animal assisted intervention strategies

WHAT IS THIS THEME ABOUT?

The focus of this theme is on the development and evaluation of accessible informal NATAIS for use in both home care and care institutions.

BACKGROUND

In addition to formal interventions, there is a pressing need for accessible and cost-effective informal NATAIS options in home care and care institutions. These strategies have the potential to impact the working environment of professional caregivers, influence the surrounding environmental stimuli and foster a sense of purpose and connection with nature. For example, in an activity that enhances both purpose and social engagement, participants collect eggs at a green farm and later enjoy eating the eggs together during a communal lunch. During particularly challenging periods marked by social isolation and restrictions on outdoor activity, alternative components may include nature sounds and visuals, guided online nature meditations, or interactive online sessions involving pets.

DEFINITONS

Informal strategies

Informal strategies encompass non-formalized behaviors and actions that clients and caregivers independently engage in, either alongside or independent of formal interventions. These strategies are typically employed when it becomes clear that an individual is experiencing certain specific challenges such as feeling lonely or depressed. For example, a relative might visit with a dog to provide their loved one with some extra companionship, or, in the context of NATAIS during the COVID-19 pandemic, a caregiver might notice a client exhibiting signs of loneliness and take that client for a walk in the park.

Environmental stimuli

Environmental stimuli can be either intentional (resulting from interventions or communication) or unintentional (e.g., hallway noises or other residents screaming). The presence or absence of environmental stimuli can lead to either over- or under-stimulation in individuals with NDD.

Purposeful activities

Purposeful activities are integrated into everyday life and serve a distinct purpose. As a result, individuals perceive them as meaningful opportunities to make a valuable contribution, as in the example of collecting eggs at green care farms to be shared with others as part of a communal breakfast.

Informal Strategies
Environmental Stimuli
Working Environment
Purposeful Activities
Accessible Interventions

CURRENT EVIDENCE AND RESEARCH GAPS

- Enhancing a relationship (e.g., adopting a welcoming attitude), increasing contact frequency, and improving communication among professional caregivers, family caregivers, and people with NDD elevates the quality of care and strengthens family caregiver involvement.
- The availability of NATAIS promotes physical exercise, alleviates stress, and encourages social interaction, all of which positively impact well-being.
- Purposeful activities that include physical activity are considered meaningful, and the freedom to use green space is empowering and enhances quality of life for people with NDD.
- Purposeful activities at care farms provide meaning and feelings of being useful.
- Environmental stimuli (e.g., textures and smells) experienced during gardening are considered valuable by people with NDD.
- Natural environments are often low in environmental stimuli, contributing to the restoration of working memory and controlled attention. This leads to better-focused attention in people with NDD.
- Caregivers can use informal strategies such as personal attention, positive approach, environmental modification, and client encouragement to motivate residents with NDD to participate in NATAIS.
- Generally, people who engage in physical outdoor activities (e.g., running or walking) in natural settings tend to experience greater happiness and reduced exhaustion compared to those who exercise indoors.
- Minimizing intentional and unintentional stimuli in nursing homes can reduce challenging behavior in residents with advanced dementia; however, it is important to consider that environmental stimuli can also be beneficial for people with NDD.
- Caregivers can actively engage people with NDD in NATAIS by assigning them purposeful activities such as collecting eggs or tending to plants.

FUTURE RESEARCH QUESTIONS

- What is the impact of accessible NATAIS on various outcomes, including domains related to well-being in people with NDD, such as autonomy, quality of life, affect, and self-worth?
- What impacts do nature-based sounds, familiar household noises (e.g., watering of plants, rustling of leaves), and management of unintentional noises (e.g., gentle music, sound scaping) have on challenging behavior in people with NDD?
- To what extent does the sense of purpose explain the effects of NATAIS?
- What are the barriers and facilitators to accessible informal NATAIS for people with NDD as perceived by these individuals themselves, their caregivers, and other stakeholders?
- What is the impact of accessible NATAIS on outcomes for informal and professional caregivers, such as their emotions, cognitions, and knowledge?
- How can NATAIS influence the working environment of professional caregivers?
- Can informally provided NATAIS account for the effects of formally provided nature-based interventions or animal-assisted interventions?
- Does the implementation of NATAIS have a positive influence on professional caregivers' experience of the work environment?
- What are the most effective strategies for developing, implementing, and evaluating accessible, informal NATAIS tailored for home care and care institutions? This includes considering their impact on professional caregivers, the sensory environment, and the cultivation of a sense of purpose and wellbeing for people with NDD, particularly in challenging circumstances such as heightened social isolation and restricted outdoor activities.

LITERATURE SOURCES

- Boumans, J., van Boekel, L. C., Baan, C. A., & Luijkx, K. G. (2018). How can autonomy be maintained and informal care improved for people with dementia living in residential care facilities: A systematic literature review. *The Gerontologist*, 59(6), e709-e30.
- De Boer, B., Beerens, H. C., Katterbach, M. A., Viduka, M., Willemse, B. M., & Verbeek, H. (2018). The physical environment of nursing homes for people with dementia: Traditional nursing Homes, small-scale living facilities, and green care farms. *Healthcare*, 6(4), 137.
- De Boer, B., Verbeek, H., Zwakhalen, S. M. G., & Hamers, J. P. H. (2019). Experiences of family caregivers in green care farms and other nursing home environments for people with dementia: a qualitative study. *BMC Geriatrics*, 19(1), 149.
- Ibsen, T. L. & Eriksen, S. (2020). The experience of attending a farm-based day care service from the perspective of people with dementia: A qualitative study. *Dementia, 20(4), 1356-1374.*
- IVN. Verwijs door naar groen. Factsheet voor artsen over het effect van de natuur op onze gezondheid. Amsterdam: Instituut voor Natuureducatie en Duurzaamheid; 2015.
- Knippenberg, I.A.H., Leontjevas, R., Nijsten, J. M. H., Bakker, C., Koopmans, R. T. C. M., & Gerritsen, D. L. (2022). Stimuli changes and challenging behavior in nursing homes during the COVID-19 pandemic. *BMC Geriatrics*, 22(1), 142.
- Knippenberg, I.A.H., Leontjevas, R., Stoyanov, S., Persoon, A., Verboon, P., Vermeulen, H., ... & Gerritsen, D. L. (2023). Informal antidepressant strategies for nursing home residents: two group concept mapping studies. *Aging & Mental Health*, 27(2), 251-62.
- Mmako, N. J., Courtney-Pratt, H. & Marsh, P. (2020). Green spaces, dementia and a meaningful life in the community: A mixed studies review. *Health & Place,* 63**,** 102344.
- Smerecnik, C., & Zwerts, L., (2014). *De kracht van groen: Een onderzoek naar de effecten van groenvoorziening op de kwaliteit van leven bij mensen met dementie.* Eindhoven: Fontys Hogeschool Psychology Ha.
- Smith-Carrier, T. A., Beres, L., Johnson, K., Blake, C., & Howard, J. (2021). Digging into the experiences of therapeutic gardening for people with dementia: An interpretative phenomenological analysis. *Dementia*, *20*(1), 130-147.
- Zhang, H., Wu, Y., Wang, N., Sun, X., Wang, Y., & Zhang, Y. (2022) Caregivers' experiences and perspectives on caring for the elderly during the COVID-19 pandemic: A qualitative systematic review. *Journal of Nursing Management*, 30(8), 3972-95.

Theme 7
Caregivers: their attitudes, needs, and training and education

Keywords:
Professional caregivers, family caregivers, needs, attitudes, skills, education, training

Authors:
Sieka Bos, Ruslan Leontjevas, Ine Declercq

Contact details:
roeslan.leontjevas@ou.nl

NATAIS
nature and animal assisted intervention strategies

WHAT IS THIS THEME ABOUT?

This theme focuses on understanding what both formal and informal caregivers need to effectively conduct NATAIS and explores their attitudes towards these intervention strategies. To effectively conduct NATAIS, caregivers need specific qualifications that can be acquired through regular education programs, specialized training, or their own experience.

BACKGROUND

By examining the perspectives of formal and informal caregivers, valuable insight can be gained into the challenges, resources, and support systems necessary to implement NATAIS effectively. Exploring their beliefs, perceptions, and opinions helps to identify potential barriers and facilitators to implementing NATAIS. By comprehending caregiver attitudes towards NATAIS, interventions can be tailored to address their concerns and enhance their acceptance and engagement. By identifying the necessary qualifications, steps can be taken to develop training programs or educational initiatives that equip caregivers with the expertise needed to effectively implement NATAIS. Research is needed to inform the development of supportive resources, tailored training programs, and strategies that address the unique challenges faced by caregivers and promote the optimal delivery of NATAIS.

DEFINITONS

Formal caregivers (professional caregivers)

Formal caregivers are trained professionals who provide help and support to clients with dependency due to disabling conditions or reduced mobility. Formal caregivers provide their clients with paid services such as personal care, household chores, meal preparation, transportation, and organizing leisure activities. Formal caregivers can work in different settings. This agenda is primarily focused on clients and caregivers in nursing homes, assisted living facilities, and private homes.

Informal caregivers (e.g., family caregivers)

Informal caregivers provide care to their family members, close relatives, friends, and neighbors. Often, this support and assistance is provided unpaid and without contracting the caregiver. Because informal caregivers contribute significantly to the well-being of individuals with illnesses and disabilities, their involvement in health care can be (financially) stimulated by governments.

Attitudes

Attitude is a complex construct that has many definitions in scientific literature. In implementation research, the concept reflects how favorably stakeholders regard practicing evidence-based interventions such as NATAIS. In general, attitudes can include cognitive, affective, and behavioral components. In terms of NATAIS, stakeholders may have positive or negative beliefs about, and emotional responses to, interventions (e.g., activities involving specific animals). The behavioral component of attitude reflects actions and intentions toward the concept of interest.

Caregivers' needs

Formal and informal caregivers have unique needs that should be addressed to enable them to provide the best care. For example, formal caregivers may need access to resources, training and education, and support from their organization and colleagues. Informal caregivers may need emotional support, assistance with specific tasks, and access to information. Meeting these needs may help caregivers with implementing NATAIS and approaches that they experience as new or alternative to regular care.

Qualification and education

Qualifications reflect skills, experience, and knowledge resulting from training or education. To ensure effective implementation of NATAIS, formal and informal caregivers can be informed about potential strategies and trained in their use. Caregivers can experience positive effects of working with NATAIS that may improve their attitudes towards these strategies.

Professional Caregivers
Family Caregivers
Needs
Attitudes
Skills
Education
Training

CURRENT EVIDENCE AND RESEARCH GAPS

- In contrast to general research on caregiver attitudes and needs, there is a lack of research specifically focused on caregiver attitudes towards NATAIS.
- Formal and informal caregivers need information on potential effects of NATAIS and on how to implement these strategies; however, to date, educational material regarding NATAIS remains lacking.
- Formal and informal caregivers may receive training aimed at improving attitudes and providing the skills and experience required for NATAIS. Currently, little is known about such initiatives.
- After the COVID-19 pandemic, educational programs and interventions for caregivers and people with NDD will likely pay greater attention to the effects of pandemics and social isolation on different outcomes. It is important to incorporate NATAIS in such educational material.

FUTURE RESEARCH QUESTIONS

- What are the needs of formal and informal caregivers for successful implementation of NATAIS in the (daily) care of people with neurodegenerative disease?
- What are the experiences with and attitudes towards NATAIS in formal and informal caregivers?
- Are there specific caregiver needs regarding NATAIS during pandemics or times of increased social isolation and loneliness?
- What are the differences in caregiver attitudes and needs in different long-term care settings (e.g., care at home and institutionalized care)?
- Which interventions can improve formal and informal caregivers' attitudes towards specific NATAIS?
- How can regular educational programs for health and social care staff be extended with learning objectives related to NATAIS?
- Which skills are needed to perform NATAIS, and what are the minimum qualifications needed?
- How to involve management in propagating NATAIS and alert them to standards of practice?
- How to develop and execute training of both formal and informal caregivers to help implement NATAIS and understand signals of adequate and inadequate care that NATAIS agents (nature-based elements and animals) receive?
- How can the perspectives of formal and informal caregivers inform the design of comprehensive training initiatives and educational resources that empower caregivers with the necessary expertise to effectively implement NATAIS, thereby bolstering the quality of support provided to individuals with NDD under challenging circumstances, such as those experienced during the COVID-19 pandemic?
-

NATAIS

LITERATURE SOURCES

- Baun, M. M., & Mccabe, B. W. (2003). Companion animals and persons with dementia of the Alzheimer's type: therapeutic possibilities. *American Behavioral Scientist*, 47(1), 42–51.
- Fishman, J., Yang, C., & Mandell, D. (2021). Attitude theory and measurement in implementation science: a secondary review of empirical studies and opportunities for advancement. *Implementation Science*, 16, 1-10.
- Fritz, C. L., Hart, L. A., Farver, T. B., & Kass, P. H. (1996). Companion animals and the psychological health of Alzheimer patients' caregivers. *Psychological Reports*, 78(2), 467–481.
- Li, J., Song, Y. (2019). Formal and informal care. In: Gu, D., Dupre, M. (Eds.) *Encyclopedia of gerontology and population aging*. Cham: Springer International Publishing.
- Parveen, S., Mehra, A., Kumar, K., & Grover, S. (2021). Knowledge and attitude of caregivers of people with dementia. *Geriatrics & Gerontology International*, 22(1), 19-25. 10.1111/ggi.14304.
- Richter, J. M., Roberto, K. A., & Bottenberg, D. J. (1995). Communicating with persons with Alzheimer's disease: experiences of family and formal caregivers. *Archives of Psychiatric Nursing*, 9(5), 279-285.

Theme 8
Technological solutions

Keywords:
Technology, artificial intelligence, pet-robots, virtual and augmented reality

Authors:
Ine Declercq, Sieka Bos, Ruslan Leontjevas

Contact details:
ine.declercq@ou.nl

NATAIS
nature and animal assisted intervention strategies

WHAT IS THIS THEME ABOUT?

This theme focuses on the role of technological solutions in providing interventions or counterbalancing challenges. Such challenges may be specific to an intervention, target group, or context and include, among others, global issues such as pandemics and environmental phenomena. It is important to harness the power of technology for optimizing the delivery, accessibility, and outcomes of NATAIS, benefiting both caregivers and people with NDD.

BACKGROUND

Over the last decade technologies such as telehealth, telemedicine, artificial intelligence, and robots have come to play an increasingly significant role in health care. Although these technologies offer many potential benefits, there is still much to learn about them, especially in relation to interventions that include nature or animals and with people with NDD. During the COVID-19 pandemic, the importance of telehealth came to be emphasized globally. Anecdotal and rarely reported evidence suggests that several different technological solutions, such as tele-counseling, video conferencing, and even virtual reality, have been used by therapists in mental healthcare who work with NATAIS; however, there is a lack of insight into how and for which clients the use of telehealth and technology can be seen as appropriate for use in NATAIS..

DEFINITONS

Telehealth & telemedicine

The terms telehealth and telemedicine are often used interchangeably. Telemedicine refers to communication between a client and therapist that takes place via videoconferencing. Telehealth refers to telemedicine, but it also refers to other applications such as remote patient monitoring, store-and-forward technologies and the use of applications in health care for mobile smartphones, wearables, and other mobile devices.

Artificial intelligence

Artificial intelligence (AI) refers to the ability of machines to learn human-like skills through predefined algorithms. It can have many applications for people with NDD (e.g., screening, diagnosing, communicating, and treating).

Social robots and robotic pets

Social robots and robotic pets are examples of AI applications. Social robots are designed to interact and communicate with people while adhering to behavioral norms. Robotic pets, on the other hand, resemble companion animals in their appearance and behavior. Some notable examples of social robots include JustoCat, Paro, and Aibo Robot Dog.

Virtual reality and augmented reality

In virtual reality (VR), a simulated three-dimensional environment provides a fully immersive experience, allowing the user to physically interact with this virtual environment. In augmented reality (AR), the user still experiences the real world, but it is enhanced with additional holograms.

Technology
Artificial Intelligence
Pet-robots
Virtual Reality
Augmented Reality

CURRENT EVIDENCE AND RESEARCH GAPS

- Telehealth and telemedicine (e.g., virtual pet visits) have shown benefits regarding the mood and well-being of clients; however, challenges remain with respect to integrating telehealth and telemedicine into daily care. For implementation purposes, staff need skills and instruments.
- Social robots and robotic pets can improve various domains of well-being for people with NDD; however, there is still a lot to learn about how interventions could be matched with the needs of people with NDD. It is not clear whether treatment effects are the same when robotic pets are used instead of real animals.
- The greatest advantage of VR and AR is that socially isolated people with NDD and those living in institutions can undergo an immersive virtual experience with nature and animals. Alongside the many positive effects on the well-being of people with NDD, there are still concerns about potential negative effects (e.g., regarding awareness, fear, and anxiety).
- Ethical concerns remain about privacy, anthropomorphizing robotic pets as living animals, and the subsequent possible attachment people may develop to these devices. In addition, pragmatic concerns can be raised about cost and user-friendliness.
- Technological solutions can be animal-friendly and useful in times of social isolation, such as during pandemics like COVID-19.

FUTURE RESEARCH QUESTIONS

- Which elements of NATAIS can be replaced by technological solutions like AR and VR, and are these replacements cost-effective and ethically justified, especially in times of social isolation?
- What are the attitudes of people with NDD and their formal and informal caregivers towards the use of technological solutions in NATAIS, and how can these attitudes be changed if needed?
- Are technological solutions as effective as real-life NATAIS in reducing loneliness and improving well-being in people with NDD and their caregivers, especially in times of social isolation?
- How can NATAIS interventions with technological solutions be fine-tuned to better address the needs of people with NDD?
- What are the negative effects of working with technological solutions in NATAIS, and how can these potential negative effects be minimized or prevented?
- How can the privacy of people with NDD and that of their professional and family caregivers be protected? Which protocols for technological solutions are justified?
- How can technological solutions in NATAIS be implemented successfully in daily care practice in different settings (e.g., home care or nursing home care)?
- How can technology effectively bridge the gap between the challenges posed by global pandemics and the delivery of NATAIS for individuals with NDD, and what are the optimal strategies for tailoring and implementing technological solutions to ensure sustained well-being and support during periods of social isolation and health crises?

LITERATURE SOURCES

- D'Cunha, N. M., Nguyen, D., Naumovski, N., McKune, A. J., Kellett, J., Georgousopoulou, E. N., Frost, J., & Isbel, S. (2019). A Mini-review of virtual reality-based interventions to promote well-being for people living with dementia and mild cognitive impairment. *Gerontology*, *65*(4), 430–440.
- Groom, L. L., McCarthy, M. M., Stimpfel, A. W., & Brody, & A. A. (2021). Telemedicine and telehealth in nursing homes: An integrative review. *Journal of the American Medical Directors Association*, *22*(9), 1784–1801.e7.
- Jung, C., Jung, C., Jongyotha, K., De, I., Brennan, M., & Naumovski, J. (2021). Using virtual pet to replace pet therapy visits in our nursing facility during the COVID-19 pandemic. *Journal of the American Medical Directors Association*, *22*(3), B18.
- Koh, W. Q., Ang, F. X. H., & Casey, D. (2021). Impacts of low-cost robotic pets for older adults and people with dementia: scoping review. *JMIR rehabilitation and assistive technologies*, *8*(1), e25340.
- Koh, W. Q., Felding, S. A., Budak, K. B., Toomey, E., & Casey, D. (2021). Barriers and facilitators to the implementation of social robots for older adults and people with dementia: A scoping review. *BMC geriatrics*, *21*(1), 351.
- Kong, C., & Soon, S. M. C. (2022). Virtual volunteering during the COVID-19 pandemic: case studies of virtual animal-assisted activities in a Singapore hospice. *Journal of Social Work in End-of-Life & Palliative Care*, *18*(3), 203–215.
- Leontjevas, R., Knippenberg, I., Bakker, C., Koopmans, R., & Gerritsen, D. (2021). Telehealth and telecommunication in nursing homes during COVID-19 antiepidemic measures in the Netherlands. *International Psychogeriatrics, 33*(8), 835-836.
- Skurla, M. D., Rahman, A. T., Salcone, S., Mathias, L., Shah, B., Forester, B. P., & Vahia, I. V. (2022). Virtual reality and mental health in older adults: a systematic review. *International psychogeriatrics*, *34*(2), 143–155.

NATAIS

Theme 9
Physical environment

Keywords: Physical environment, green space, landscaping, interior design, social interaction, walking-friendly neighbourhood, public open space

Authors:
Ingeborg Pedersen, Peter Reniers, Sofie Hoorelbeke, Yvonne van der Leest, Debby Gerritsen

Contact details:
ingeborg.pedersen@nmbu.no

NATAIS
nature and animal assisted intervention strategies

WHAT IS THIS THEME ABOUT?

This theme centers on the designing of environments that nurture well-being, alleviate loneliness, and foster social connectedness in people with NDD. Through the integration of NATAIS elements and consideration of global challenges such as pandemics and climate change, environmental design can encompass strategies that emphasize sustainability, climate adaptability, health and well-being, social equity, and inclusivity. This approach facilitates the development of spaces that effectively support the immediate needs of people with NDD.

BACKGROUND

By integrating scientific evidence into the environmental design process, health care environments can be optimized to cater to the distinctive needs of people with NDD. Careful landscaping can create soothing and engaging outdoor spaces encompassing features such as sensory gardens, nature trails, or outdoor seating areas that promote relaxation, sensory stimulation, and a connection with the natural world, thereby facilitating NATAIS.

Architectural design considerations that embrace NATAIS may involve designing layouts that facilitate easy navigation and accessibility to outdoor spaces. This could include incorporating living green walls, indoor gardens, or natural elements such as wood or stone. In terms of interior design choices, suitable furnishings and decor can contribute to a serene atmosphere, reduce sensory overload, and provide comfortable and functional spaces for various activities. For instance, soft lighting, comfortable seating areas adorned with plants, and strategically placed sensory materials or interactive elements can enhance NATAIS experiences.

DEFINITIONS

Physical environment

The physical environment encompasses landscapes, architecture, and interior design. Landscaping refers to the organization, layout, and enhancement of outdoor spaces, including gardens, parks, or yards. Architecture involves the design and construction of buildings, while interior design is dedicated to creating and arranging functional and visually appealing interior spaces within buildings.

Physical Environment
Green Space Landscaping
Interior Design
Social Interaction Walking-friendly neighbourhood
Public Open Space

CURRENT EVIDENCE AND RESEARCH GAPS

- Several factors in the physical environment are known to promote the use of gardens in health care facilities and green public open spaces. These include easy access, regardless of season or weather conditions, wheelchair-friendly garden paths, clear way-finding systems, varied seating options, sheltered areas, and sensory-stimulating plants that display seasonal changes and attract insects and birds.
- Resident satisfaction and engagement with the outdoor space may be enhanced through collaborative design of green gardens with input from people with NDD, family caregivers, and professional caregivers.
- For people with NDD living at home, the outdoor environment can be both supportive and potentially anxiety-inducing. Spending time outdoors can offer physical and social opportunities and contribute to everyday activities, all of which can have therapeutic benefits.
- Public open spaces may also provide similar therapeutic benefits by offering opportunities for physical, social, and everyday activities.
- For people with NDD, the opportunity to experience autonomy is an important factor when considering the use of public open spaces for physical and social activities.
- Older adults may experience benefits from engaging in activities in public open spaces, in terms of both physical and mental well-being.
- Outdoor communal spaces at residential facilities have the potential to foster a sense of social inclusion, especially in times of social isolation, such as during the COVID-19 pandemic.

FUTURE RESEARCH QUESTIONS

- What effects do indoor and outdoor areas, landscaping, and interior design have on health outcomes in people with NDD?
- How can the physical (care) environment help prevent social isolation and improve quality of life in people with NDD?
- In what ways can the physical environment stimulate and facilitate physical activity among people with NDD?
- How can the physical environment improve the sleep quality of people with NDD?
- What is the relationship between the utilization of different spaces and social interaction among people with NDD?
- What is the connection between observing the outdoor environment from an indoor space (e.g., through a window) and overall well-being?
- What is the relationship between co-designed gardens and quality of life in people with NDD?
- What associations exist between specific characteristics of public open spaces and both physical and social activities of people with NDD?
- How can the principles of environmental design be effectively employed to address the challenges presented by global pandemics?
- How can spaces be optimized to cater to the immediate needs of people with NDD amidst the evolving dynamics of health crises?

LITERATURE SOURCES

- Astell-Burt, T., Hartig, T., Putra, I. G. N. E., Walsan, R., Dendup, T., & Feng, X. (2022). Green space and loneliness: A systematic review with theoretical and methodological guidance for future research. *Science of the Total Environment*, *847*, 157521.
- Brittain, K., Corner, L., Robinson, L., & Bond, J. (2010). Ageing in place and technologies of place: The lived experience of people with dementia in changing social, physical and technological environments. *Sociology of Health & Illness*, 32(2), 272-287.
- Charras, K., Be'bin, C., Laulier, V., Mabire, J-B., & Aquino, J-P. (2020). Designing dementia-friendly gardens: A workshop for landscape architects: Innovative Practice. *Dementia*, 19(7), 2504–2512.
- Faris, S., Stigsdotter, U., & Nilsson, K. (2012). A review of design recommendations for outdoor areas at healthcare facilities. *Journal of Horticultural Therapy*, 22(2), 33-47.
- Gibson (2018). “Let's go to the park.” An investigation of older adults in Australia and their motivations for park visitation. *Landscape and Urban Planning,* 180, 234-246.
- Giebel, C., de Boer, B., Gabbay, M., Watkins, C., Wilson, N., Tetlow, H., & Verbeek, H. (2022). Developing a meaningful garden space in a care home with staff and family members: A qualitative study. *International Journal of Environmental Research and Public Health*, 19(12), 7025.
- Levy-Storms, L., Chen, L., & Loukaitou-Sideris, A. (2018). Older adults' needs and preferences for open space and physical activity in and near parks: A systematic review. *Journal of Aging and Physical Activity*, 26 (4), 682-696.

NATAIS

Theme 10
Effects of different types of NATAIS in people with NDD

Keywords:
Wel-lbeing, quality of life, loneliness, short-term effects, long-term effects

Authors:
Mascha Molog, Mayke Janssens, Dorit van Meel, Simone de Bruin, Birgitta Erixon Halck

Contact details:
mascha.molog@ou.nl

NATAIS
nature and animal assisted intervention strategies

WHAT IS THIS THEME ABOUT?

This theme revolves the (therapeutic) effects of different types of NATAIS. The primary outcomes considered encompass general well-being, quality of life, and the impact on loneliness. While well-being and quality of life serve as overarching indicators of positive outcomes, the inclusion of loneliness is crucial in times of increased social isolation, such as during the COVID-19 pandemic.

BACKGROUND

Nature and Animal-Assisted Intervention Strategies (NATAIS) refer to non-pharmacological strategies that can have many positive effects on outcomes in people with NDD. These may include improved quality of life, enhanced social behavior and decreased agitation, loneliness, and depression. Within each type of strategy, elements vary widely, including intervention length, type of animal, type of nature-based element, and type of activity. For example, activities may be conducted in the presence of others, with social interaction or other non-pharmacological elements such as activities including music or exercise. Although promising results have already been found, more research is necessary. There is still a shortage of empirical evidence to inform the practitioners and other research-consumers who develop or use NATAIS.

DEFINITONS

Therapeutic effect

Therapeutic effect refers to the beneficial outcomes of a specific intervention, treatment, or therapy. The assessment and measurement of therapeutic effects are crucial in evaluating the efficacy and effectiveness of interventions in various fields, including health care psychology, and rehabilitation.

Outcomes

An outcome is the result or consequence of something. Outcomes of interventions, therapies, or activities may reflect the desired changes, improvements, or relief experienced by individuals. Examples of outcomes may include enhanced quality of life; improved functioning; better sleep; decreased loneliness and social isolation; fewer symptoms; less challenging behavior; and any other positive changes related to the targeted condition or goals of the therapy.

Well-being
Quality of Life
Loneliness
Short-term Effects
Long-term Effects

CURRENT EVIDENCE AND RESEARCH GAPS

- Research has shown beneficial effects of animal-assisted interventions (AAIs) on quality of life, physical activity, agitation, communication skills, eating habits, loneliness, depression, anxiety, aggression, apathy, cognitive impairment, and social behavior in people with NDD; however, several studies showed no significant improvements in activities of daily living, cognitive impairment, and quality of life following AAI in people with NDD.
- Nature-based activities such as spending time in a garden, showed positive effects in people with NDD living in nursing homes. These included improved sleep patterns and quality of life; stimulated senses; increased social interaction, physical activity and engagement in activities; and reduced agitated behavior and feelings of depression. Little is known about the impact of nature-based activities for people with NDD in the community.
- Green care farms showed effects in people with NDD on spending time outdoors, including improved engagement in everyday activities; increased physical activity; improved social health and social participation; healthy eating; enhanced feelings of autonomy and meaning in life—however, research on the long-term impact of living at care farms is still scarce.
- Interest in blue health is increasing, and recent research suggests that the presence of water can enhance health and well-being, but research in this field is still scarce.

FUTURE RESEARCH QUESTIONS

- What are the long-term effects of different types of NATAIS in people with NDD?
- What is the dose-response of NATAIS (e.g., do the effects improve with the number of sessions)?
- Do the effects of different types of NATAIS on outcomes in people with NDD depend on the type of NDD, severity of the disease, and age?
- What is the relative effect of NATAIS compared to other accepted non-pharmacological interventions, such as music or art therapy?
- Can the effect of different types of NATAIS be enhanced by combining them with other interventions (e.g., medication)?
- What are the effects of different types of NATAIS in people with NDD living in the community?
- Which side effects can be found for different types of NATAIS?
- How can different types of NATAIS counter the negative effects of pandemic restrictions, such as those imposed during the COVID-19 pandemic, in people with NDD?

LITERATURE SOURCES

- Batubara, S. O., Tonapa, S. I., Saragih, I. D., Mulyadi, M., & Lee, B. O. (2022). Effects of animal-assisted interventions for people with dementia: A systematic review and meta-analysis. *Geriatric Nursing*, *43*, 26-37.
- Barrett, J., Evans, S., & Mapes, N. (2019). Green dementia care in accommodation and care settings: a literature review. *Housing, Care and Support*, *22*(4), 193-206.
- De Boer, B., Hamers, J. P., Zwakhalen, S. M., Tan, F. E., Beerens, H. C., & Verbeek, H. (2017). Green care farms as innovative nursing homes, promoting activities and social interaction for people with dementia. *Journal of the American Medical Directors Association*, *18*(1), 40-46.
- De Bruin, S. R., Stoop, A., Molema, C. C., Vaandrager, L., Hop, P. J., & Baan, C. A. (2015). Green care farms: An innovative type of adult day service to stimulate social participation of people with dementia. *Gerontology and Geriatric Medicine*, *1*, 2333721415607833.
- Hu, M., Zhang, P., Leng, M., Li, C., & Chen, L. (2018). Animal-assisted intervention for individuals with cognitive impairment: A meta-analysis of randomized controlled trials and quasi-randomized controlled trials. *Psychiatry Research*, *260*, 418-427.
- Marks, G., & McVilly, K. (2020). Trained assistance dogs for people with dementia: A systematic review. *Psychogeriatrics*, *20*(4), 510-521.
- Mmako, N. J., Courtney-Pratt, H., & Marsh, P. (2020). Green spaces, dementia and a meaningful life in the community: A mixed studies review. *Health & Place*, *63*, 102344.
- Motealleh, P., Moyle, W., Jones, C., & Dupre, K. (2019). Creating a dementia-friendly environment through the use of outdoor natural landscape design intervention in long-term care facilities: A narrative review. *Health & Place*, *58*, 102148.
- Smith-Carrier, T. A., Beres, L., Johnson, K., Blake, C., & Howard, J. (2021). Digging into the experiences of therapeutic gardening for people with dementia: An interpretative phenomenological analysis. *Dementia*, *20*(1), 130-147.
- Zafra-Tanaka, J. H., Pacheco-Barrios, K., Tellez, W. A., & Taype-Rondan, A. (2019). Effects of dog-assisted therapy in adults with dementia: A systematic review and meta-analysis. *BMC psychiatry*, *19*(1), 1-10.

NATAIS

NATAIS
nature and animal assisted
intervention strategies

Financed by

GHENT UNIVERSITY

UKON Universitair Kennisnetwerk Ouderenzorg Nijmegen

Open Universiteit

JPND research
EU Joint Programme – Neurodegenerative Disease Research

fwo
