# Supplementary material for: Developing a research agenda on NATure-based and Animal-assisted Intervention Strategies (NATAIS) in people with neurodegenerative diseases with a specific focus on social isolation and loneliness: a group concept mapping procedure
Source: BMC Geriatr. 2024 Sep 28;24:795. doi: 10.1186/s12877-024-05387-2 (PMC11439302; doi:10.1186/s12877-024-05387-2)
Supplement: Supplementary file 2 — Additional file 2. [file 12877_2024_5387_MOESM2_ESM.pdf]

Table 1

Results of the group discussion

Cluster	Initial Cluster Name (Fig 2)	Cluster Name after discussion (Fig. 3)	Initial content of the chapter	Research ideas and or statements
**1**	Insight into crucial components and working mechanisms	Theoretical underpinnings and working mechanisms<br><br>(Theme 2 in the research agenda)	Wat are the crucial components to reduce social isolation among people with neurodegenerative diseases and how can we match the working elements in NATAIS better to these components to reduce loneliness?	• Effects of Green Care/AAI compared to other highly accepted interventions<br>• Effects on different outcomes of a green environment (e.g., urban greenery, wild greenery, greenery) compared to settings without plants/animals/views of nature<br>• Differences regarding the preference of different groups in green care elements (green nature, activities with animals etc.)<br>• Generalizability of green care elements to different types of dementia<br>• Effects of nature elements on different outcomes (e.g., stress)<br>• Insight into crucial components to lower social isolation (e.g., is 'talking to' crucial?)<br>• Dose-response relationship and long term effects of interventions in the field of green care/AAI and their effects in people with cognitive impairments<br>• Interactions between vision and other sensories when green care elements are present, e.g. vegetation versus animals<br>• Insight into the role of reminiscence in green care (e.g., are people more attracted to different types of landscapes that can be related to the person's history/past?)<br>• More insight into biophilia hypothesis (what attracts clients more, living beings (animals, people), or green nature)<br>• Insight into the working elements of Green Care/AAI (e.g., rest, relaxation, pleasure, release of oxytocin to reduce stress)<br>• Match working elements in Green Care/AAI to outcomes (e.g., match working elements to reduce loneliness)

**2**	Matching NATAIS to needs of different target groups	Merged with cluster 3: Identifying target groups and matching NATAIS to their needs and preferences<br><br>(Theme 3 in the research agenda)	What works for whom and how to evaluate the outcome effects and experiences in the different target populations (e.g., differences between neurodegenerative diseases, professional or family caregivers, ethnicity etc.)?	• Alignment of green care with the needs and capacities of people with dementia (and their carers), taking into account dementia stage. For instance, the need to nurture.<br>• Measurement tools to evaluate effects of green care/AAI interventions<br>• Differences regarding the benefits of different groups in green care interventions (cultural background, e.g., ethnicity, SES, gender, religion)<br>• Cultural diversity and inclusion*(9)<br>• Peoples' experiences of a green environment (e.g., meaningful)<br>• Effects of green care on caregivers (professional or family caregivers) of people with neurodegenerative disorders (e.g., effect on their Quality of life)
**3**	Goal-oriented NATAIS	Merged with cluster 2: Identifying target groups and matching NATAIS to their needs and preferences<br><br>(Theme 3 in the research agenda)	How to improve NATAIS to be able to use interventions more goal-oriented and, subsequently, get better treatment outcomes?	• Insight into the working elements of Green Care/AAI (e.g., active versus passive interaction, approaching actively, or being approached by)<br>• Insight into the working elements of Green Care/AAI (e.g., which sensories to stimulate, synchronisation, interaction, attention, approaching animals actively or being approached by)<br>• Green environment and the presence of animals to promote different activities (e.g., physical activities, stimulate communication)<br>• Green Care/AAI to promote different activities (e.g., activities like walking, caretaking of the animals)<br>• Insight into the working elements of interventions (e.g., individually or in group)<br>• Match working elements in Green Care/AAI to patients (e.g., different approaches for different diagnosis?)<br>• Combining different Green Care activities to create more effective forms (e.g., a combination of AAT and green exercise)<br>• Combine green care with other types of care interventions *(11)

**4**	Insight into the positive effects of NATAIS	Effects of different types of NATAIS in people with NDD<br><br>(Theme 10 in the research agenda)	Effects of NATAIS on different outcomes and how can these positive effects protect people with neurodegenerative diseases and their social environment?	• Long-term impact of green care on people with dementia (longitudinal studies on sleeping patterns, end-of-life processes, delay of institutionalization, relationships with family and friends, participation in society etc.)<br>• Effects of green care/AAI on challenging behaviour in people with dementia<br>• Effects of green care/AAI on relationships of people with dementia and their (in)formal carers<br>• Effects of green care/AAI on wellbeing of people with dementia and their (in)formal carers<br>• Compare effects of AAI/ horticulture therapy/care farming in clients with different diagnoses.<br>• Experiences (qualitative data, survey) of people with dementia before and after Green Care interventions/AAI<br>• Measurement tools (proxy-based, observations, technical solutions) to assess the experiences with nature and animals<br>• Effects of green care/AAI to counter the negative effects of restrictions<br>• Experiences (qualitative data, survey) of people with dementia when exposed to different types of nature elements/animals/environments<br>• Effects of caring for animals (plants) compared to the effects of receiving attention from humans and animals<br>• Effects of interaction with animals compared to interaction with humans on different outcomes (e.g., social isolation)<br>• Role of pets for people after a dementia diagnosis<br>• Effects of white noise on different outcomes (sounds that can be found in nature, like the sound of wind, sea etc.)<br>• Activities in Green Care/AAI to enhance feelings of self-worth (e.g., caretaking will enhance feelings of being 'useful', meaningful)<br>• Effects of green environment on different outcomes (e.g., cognition, problem behavior, quality of life)
**5**	Implementation in care institutions	Implementation of NATAIS	How can NATAIS be implemented	• Organizational facilitators to include NATAIS in existing care institutions.

		(Theme 4 in the research agenda)	in a way that stimulates integration of NATAIS in the community (both intramural and extramural)?	• Overcome organizational barriers to include NATAIS (i.e., acceptable limits that an institution can apply)<br>• Integration of NATAIS (as a norm - not an 'extra')<br>• Organizational barriers to include NATAIS in existing care institutions.<br>• Attitudes (of care organizations, care workers, clients) towards NATAIS<br>• High profile awards for centers of excellence (e.g., Encourage site visits/ interns)<br>• Incorporate peoples' own pets in interventions and professional support (e.g. care, social work) and its effectiveness*(9)<br>• How can you make the community ready for the integration of NATAIS (think of NDD friendly social environment), involvement of the community**
**6**	Professional skills and qualifications	Merged with cluster 7: Caregivers: their attitudes, needs, and training and education<br><br>(Theme 7 in the research agenda)	What are the qualifications for providing NATAIS and how to implement and optimize training for professional carers?	• Incorporate green care/AAI in training programs for healthcare professionals<br>• Training of competences and qualifications of care professionals to provide NATAIS in dementia care<br>• Minimum qualifications and standards for those working in NATAIS<br>• Education of gatekeepers (in care organizations) to alert them to standards of practice.<br>• NATAIS and its applications should be in the curricula of all training in the health and social care professions - even if just an introductory lecture. Post grad training should also be encouraged<br>• Realization of exchanging skills in green care**<br>• What skills are needed to perform NATAIS?**<br>• What is the situation now, what are the skills and implications for animals and nature?**
**7**	Needs of professionals/ carers	Merged with cluster 6: Caregivers: their attitudes, needs, and training and education	What do formal and informal carers need and what are their experiences and attitudes with	• Influences of a pandemic on the needs of professionals in NATAIS<br>• Pro's and con's for allowing companion animals in nursing homes => from the perspective of attitude<br>• Insight in factors that play a role in carers' experiences with respect to companion animals<br>• Experience of carers with respect to companion animals (e.g., animals as support or rather a hassle?)

		(Theme 7 in the research agenda)	respect to NATAIS?	• Carers need to understand signals of plants not receiving adequate care and act appropriate (i.e., sensitive and timely)<br>• Carers need to understand signals of animals not receiving adequate care and act appropriate (i.e., sensitive and timely*[8]<br>• Interventions to 'increase' attitudes of professional carers towards NATAIS*[6]<br>• Specific needs of professions that are involved in providing NATAIS in people with NDD*[6]
**8**	Technological solutions	Technological solutions<br><br>(Theme 8 in the research agenda)	How can technological solutions provide support in executing NATAIS, or counterbalance some of the challenges regarding NATAIS?	• Technical solutions for providing NATAIS (e.g., AI, VR, AR) in time of pandemics and beyond pandemics<br>• Institutionalized and other people with neurodegenerative disorders should have access to nature and animals by virtual reality<br>• Technical solutions to stimulate sensories (e.g., tactile, sounds, vision)<br>• Effects of technical solution, like VR, on people with neurodegenerative disorders (e.g., effects on level of awareness)*[13]<br>• Ethical issues regarding technical solutions, attitudes*[14]<br>• Alternatives for specific green care elements (e.g., nature, greenery, animals) *[9]<br>• Needs for technological solutions, when and for whom?**<br>• Animal friendlessness of technological solutions**<br>• The most important question: do we know that 'real' NATAIS are better than technological solutions**
**9**	Rest category	Methodological challenges<br><br>(Theme 5 in the research agenda)		*Statements from this cluster were divided into other clusters. However, it was agreed on that an additional cluster regarding methodological challenges was currently missing but highly needed for the research agenda.*
**10**	Research into accessible NATAIS	Research on accessible informal NATAIS	Develop and evaluate NATAIS that are accessible, easy to implement, and	• Develop and evaluate easily implementable NATAIS (i.e., small, easy to implement, at low costs)<br>• Interventions to include NATAIS at low costs at home<br>• Encourage clients to care for plants and animals when possible<br>• Usability and applicability of specific green care elements*[11]

		(Theme 6 in the research agenda)	at low costs/low profile, into home care and/or in care institutions.	• Support, and monitor clients progressively more to care for plants and animals<br>• Implementation research on feasibility, acceptability etc.
**11**	Physical environment	Physical environment<br><br>(Theme 9 in the research agenda)	Design a physical environment that contributes to wellbeing, i.e. landscaping, building and interior design.	• Building design / architectural solutions to include nature elements in daily care in nursing homes*(10)<br>• Including appropriate artwork in institutions - exceptional landscapes, accurate representations of a range of species - plants, animals and birds*(10)<br>• Representation of the changing seasons (especially important for long term residents of facilities)<br>• Choose outdoor/indoor plants carefully - suited to the environment, and with contrasting appearances and behavioral characteristics (E.g., variations in size, shape of leaves, color, flowers, climbers, creepers)<br>• Intercultural differences regarding the use of green care elements (green nature, activities with animals etc.)***(overarching)<br>• Realization of reciprocity in green care***
**12**	Negative aspects of NATAIS	Merged with cluster 13 and 14: Challenges, risk factors, ethics, and animal welfare in NATAIS<br><br>(Theme 1 in the research agenda)	What are the challenges related to NATAIS in people with neurodegenerative disease and how to measure and assure animal welfare?	• Matching animals to the environment, client and treatment outcomes (i.e., clear job descriptions for the animals in relation to therapeutic goals)<br>• Validated instruments for animal welfare in working animals, considering short and long term (cumulative) effects<br>• Insight into the potential negative effects of NATAIS on animals, environment and clients
**13**	Risk Factors	Merged with cluster 12 and 14: Challenges, risk factors, ethics,	What risk factors are associated with NATAIS and	• Green farms as instrument to help to mitigate the more general effects of the current pandemic and possible future pandemics (solution)<br>• Burdens or negative effects that arise or are intensified in human-animal relationships in times of crisis, e.g., in the pandemic<br>• The potential contribution of NATAIS to the spread of pandemic diseases

		and animal welfare in NATAIS<br><br>(Theme 1 in the research agenda)	how to overcome these risk factors?	• What are the risks associated with animal assisted interventions?<br>• The contribution of animals to the spread of pandemic diseases***<br>• The contribution of a green environment to the spread of pandemic diseases***<br>• How to mitigate these risks through careful planning, implementation, and careful assessment of animals and clients?**<br>• How to be prepared with alternative activities in case of pandemics (like alternatives for physical contact etc.)**
**14**	Ethical issues	Merged with cluster 12 and 13: Challenges, risk factors, ethics, and animal welfare in NATAIS<br><br>(Theme 1 in the research agenda)	Ethical issues related to NATAIS and how to assess physical and emotional wellbeing of animals/animal welfare?	• Animal welfare in the context of Animal Assisted Interventions<br>• Assessment of animal welfare in local setting; by knowing behaviors and emotional recognition<br>• Ethical issues to work with animals<br>• Examine / review the work schedule of the animals, e.g., hours of work, travel, ….**<br>• Examine the living and working environment conditions of animals**<br>• For resident animals, vet should visit the animals nx a year; To safely involve animals, and prevent the destruction of green environment**<br>• How many times the vet should be involved?**<br>• Who should be involved? (it's about the professionals involvement; ethologists; vets)**<br>• Distressing to see dead plants**<br>• No exotic and wild species, wild birds, should be involved in NATAIS, except for observation in a natural habitat.**

Note: The table represents the clusters and statements after the plenary group discussion. However, it is possible that research ideas were reorganized into another theme upon the decision of the responsible subgroup members after this discussion. The final research agenda, with the finalized research ideas, was agreed upon by every working group member.

*(number of initial cluster) Statements regrouped from another cluster

**Statements added during the plenary group discussion

***Statements removed during the plenary group discussion
